# Revealing the potential transmission route of Cnaphalocrocis medinalis granulovirus capable of persistently causing granulosis epidemics

**DOI:** 10.1093/ve/veaf055

**Published:** 2025-07-25

**Authors:** Baoding Chen, Yachao Zuo, Yanrong Lv, Hao Zhang, Jiawen Yang, Yun Gu, Junxiong Yang, Meijin Yuan, Kai Yang

**Affiliations:** State Key Laboratory of Biocontrol, School of Life Sciences, Sun Yat-sen University, No. 135, Xingang Xi Road, Guangzhou 510275, China; State Key Laboratory of Biocontrol, School of Life Sciences, Sun Yat-sen University, No. 135, Xingang Xi Road, Guangzhou 510275, China; State Key Laboratory of Biocontrol, School of Life Sciences, Sun Yat-sen University, No. 135, Xingang Xi Road, Guangzhou 510275, China; State Key Laboratory of Biocontrol, School of Life Sciences, Sun Yat-sen University, No. 135, Xingang Xi Road, Guangzhou 510275, China; State Key Laboratory of Biocontrol, School of Life Sciences, Sun Yat-sen University, No. 135, Xingang Xi Road, Guangzhou 510275, China; State Key Laboratory of Biocontrol, School of Life Sciences, Sun Yat-sen University, No. 135, Xingang Xi Road, Guangzhou 510275, China; State Key Laboratory of Biocontrol, School of Life Sciences, Sun Yat-sen University, No. 135, Xingang Xi Road, Guangzhou 510275, China; State Key Laboratory of Biocontrol, School of Life Sciences, Sun Yat-sen University, No. 135, Xingang Xi Road, Guangzhou 510275, China; State Key Laboratory of Biocontrol, School of Life Sciences, Sun Yat-sen University, No. 135, Xingang Xi Road, Guangzhou 510275, China

**Keywords:** baculovirus, CnmeGV, genetic structure, heterozygosity, biological control

## Abstract

As crucial regulators of insect populations in nature, baculoviruses are promising biopesticides. However, due to the scarcity of individuals with overt disease and the sporadic nature of the epidemic, our knowledge of baculovirus ecology is very limited, which impacts the effective utilization of these viruses in biocontrol. Cnaphalocrocis medinalis granulovirus (CnmeGV) specifically infects the rice leaffolder, which is the main pest of rice. In this study, we identified a population of CnmeGV that can cause a persistent epizootic in Dahuai town, Enping County, Guangdong Province, China. We sequenced the whole genomes of 138 CnmeGV isolates collected from Dahuai town for four years, reporting for the first time the genetic structure of a natural population of baculovirus. The results indicated that a long-term endemic population of CnmeGV displayed substantial genetic variation. The discriminant analysis of principal components revealed that the genetic structure of CnmeGV is clearly differentiated annually and seasonally (by the rice-growing season). CnmeGV epidemics typically occur in three waves (W1, W2, and W3) during each rice-growing season. Although the genetic structures of the CnmeGV isolates within the same rice-growing season were closely related, nucleotide diversity analysis revealed that the CnmeGV genomes exhibit higher heterozygosity levels in the initial epidemic wave compared to subsequent waves. We also found that host behaviour, virus distribution, plant structure, and weather are important factors in the recurrence of CnmeGV epizootics. Leveraging these ecological insights, we revealed the potential transmission route of CnmeGV, named ‘From W1 in sheath to W2+ in fold’, during continuous epidemics in natural environments. This study provides important insights into the ecology and evolution of host–pathogen interactions and the route helps develop more effective biocontrol strategies.

## Introduction

Baculoviruses have been utilized as effective and sustainable biological insecticides for controlling pest populations in forests and crops (Moscardi 1999, Moscardi et al. 2011, Gelaye and Negash 2023) because of their narrow host range, high virulence and safety for handlers, animals, the food supply and the environment (Haase et al. 2015). The best examples are *Anticarsia gemmatalis* multiple nucleopolyhedrovirus in soybean crops and Cydia pomonella granulovirus in fruit crops (Moscardi 1999, Oliveira et al. 2006, Abd-Alla et al. 2020). Baculoviruses are characterized by circular double-stranded DNA packaged within a rod-shaped capsid, which is enclosed by a lipid envelope (Harrison et al. 2018). All baculoviruses can survive epidemics in the form of occlusion bodies (OBs), in which virions are embedded within a proteinaceous matrix and thus remain infectious outside the host (Raymond et al. 2005). After the death of insect larvae infected with viruses, OBs accumulate in the soil, which can then be redistributed to plants through rain splash, soil disturbance, and seedling growth (Olofsson 1988, Fuxa et al. 2001, Fuxa 2008) and infect insect hosts through the ingestion of contaminated food plants by insects (Williams et al. 2017).

The family *Baculoviridae* comprises a diverse group of insect-specific viruses, and members of this family are found in more than 600 insect species (Rohrmann 2019). Despite the widespread presence of baculoviruses, they are rarely detected in most host populations, and larvae with symptoms of viral infection are even rarer (Cory and Myers 2003, Myers and Cory 2016). Although baculoviruses sometimes cause severe epizootics and significantly reduce the population densities of forest- and crop-defoliating pests in some natural systems, these outbreaks are short-lived and intermittent (Cory and Myers 2003, Myers and Cory 2016). Consequently, long-term field datasets regarding baculoviruses in natural populations remain extremely limited (Cory and Myers 2003, Sun et al. 2006, Myers and Cory 2016). Host insects, plants, the virus itself, and certain abiotic factors may contribute to viral epizootics, but many theories on the epizootics of baculoviruses and lepidopterans have been developed via mathematical models (Dwyer et al. 1997, Cory and Myers 2003, Sun et al. 2006, Elderd 2013, Paez and Fleming-Davies 2020). Recent studies have focused on the genetic diversity of one or several geographically distinct baculovirus isolates of the same species (Larem et al. 2019, Fan et al. 2021). Although baculoviruses, like their insect hosts, exist as populations and can be studied from an ecological perspective, our understanding of their ecology remains very limited (Fuxa 2004).

Rice (*Oryza sativa* L.) is a crucial cereal crop and a staple food for nearly half of the world’s population (Hegde and Hegde 2013). Rice leaffolder (RLF), *Cnaphalocrocis medinalis* Güenée, is a significant migratory pest of rice (Khan et al. 1988, Kawazu et al. 2001) and is widely distributed across Asia (Hill 1983). Approximately one month is needed for a generation of RLF to progress through four stages: egg, larva, pupa and adult (Zhang and Gu 1989, Zhang and Chen 1991). In general, the RLF has five larval instars (Xu et al. 2011). With the exception of the first instar, the higher instars longitudinally roll up rice leaves to form leaf folds and feed on the mesophyll tissue inside, which leads to a substantial reduction in rice photosynthesis and yield (Jong 1992, Wancai et al. 2016). Studies have revealed that RLF can cause rice yield losses ranging from 63% to 80% (Murugesan and Chelliah 1987). The frequent occurrence of RLF has prompted farmers to overuse chemical insecticides, which pose serious hazards to the environment and human health (Damalas and Koutroubas 2016). Thus, the management of RLF through the application of biopesticides is gaining increasing attention.

**Figure 1:**
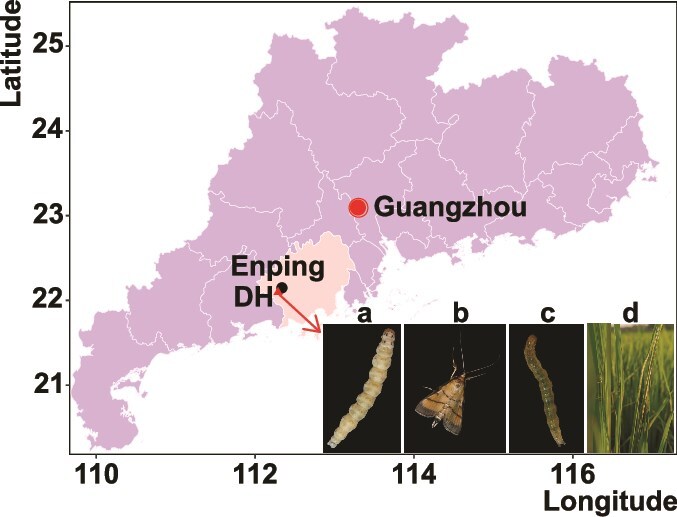
Map of the collection site for the CnmeGV isolates in this study. Samples were collected from rice fields in Dahuai town, Enping County, Guangdong Province, China, in 2017, 2018, 2019, and 2021. Panel (a) shows a CnmeGV-infected larva with a milky white body color, (b) shows an adult C. medinalis individual, (c) shows a healthy larva with a green and transparent body color, and (d) shows longitudinal rolls of rice leaves made by RLF larvae that fed on the mesophyll tissue, resulting in white leaves. The sample collection time and quantity information are provided in Section A of the S1 Data.

Cnaphalocrocis medinalis granulovirus (CnmeGV) is assigned taxonomically to the family *Baculoviridae*, genus *Betabaculovirus*, species *Betabaculovirus cnamedinalis* (Harrison et al. 2018). CnmeGV is pathogenic to RLF larvae (Zhang et al. 2015) and most RLF larvae die within 5 to 10 days after ingesting CnmeGV OBs. Although it is difficult to find CnmeGV-infected RLF larvae in natural populations, CnmeGV epizootics occur almost every year in Dahuai town, Enping County, Guangdong Province, China (Pang et al. 1981, Zhang et al. 2014, Zuo et al. 2024) (Fig. 1). It is difficult for the RLF to overwinter in this area. The first generation of RLFs migrates annually from the Indo-China Peninsula, mainly from mid-April to mid-May. After six to eight generations, the population migrates to the Indo-China Peninsula in November for overwintering (Zhang et al. 1980, Liu et al. 2020). In Dahuai town, double-cropped rice is planted annually. First, early rice is planted (from early April to mid-to-late July). After harvesting, the field is ploughed. Then, late rice is planted (from early August to late October). During each rice-growing season, CnmeGV epidemics typically occur in three successive waves. In general, the first wave (W1) occurs at the beginning of tillering and corresponds to the outbreak in the second generation of *C. medinalis* larvae; the second wave (W2) occurs at the late tillering stage and is associated with the third larval generation, and the third wave (W3) occurs at the grain filling stage, corresponding to the fourth larval generation.

This study aimed to explore the genetic variation patterns in a natural baculovirus population to help us understand the evolutionary changes and ecological impacts of these diseases and lay the foundation for the development of baculovirus-based biocontrol strategies. In this study, we monitored a CnmeGV outbreak in Dahuai town for 4 years. Using genomic data from natural populations, we described the genetic structure of CnmeGV populations and their annual variation characteristics and elucidated the nucleotide diversity pattern of CnmeGV within an epidemic season. We found that CnmeGV clearly differentiated annually and seasonally (by the rice-growing season). Although the genetic structures of the CnmeGV isolates within the same rice-growing season were closely related, nucleotide diversity analysis revealed that the CnmeGV genomes exhibit higher heterozygosity levels in the initial epidemic wave compared to subsequent waves. Most importantly, we revealed the potential transmission route of CnmeGV, named ‘From W1 in sheath to W2+ in fold’, during continuous epidemics in natural environments, which provides important insights into the ecology and evolution of host–pathogen interactions and helps develop more effective biocontrol strategies.

## Materials and methods

### Collection of isolates

The sampling location was Dahuai town (112.273° N; 22.138° E), Enping County, Guangdong Province, China. Between 2017 and 2021, we collected larvae from *C. medinalis* populations in the same rice field located in Dahuai town (Fig. 1; Section A of Supplementary Data 1). On each sampling day, we systematically collected folded rice leaves using a roughly Z-shaped random sampling route in the paddy field. The collected folded leaf samples were placed in a fresh-keeping bag containing wet paper towels. After being brought back to the laboratory, the folded leaves were peeled open and the larvae were collected. Larvae infected with CnmeGV that become sick or die are milky white in colour and can generally be identified visually (Fig. 1). However, in cases of uncertainty, we tested the larvae for the presence of CnmeGV by PCR. The virus-killed cadavers were individually transferred to a 1.5-ml centrifuge tube and stored at −20°C. Owing to the quarantine measures imposed during the COVID-19 pandemic, we did not conduct sampling in 2020.

### Baculovirus sequencing

Each isolate consisted of the OBs of CnmeGV purified from a single infected larva. The purification of OBs, extraction of viral DNA, and establishment of sequencing libraries were performed as described previously (O'Reilly et al. 1992, Zhang et al. 2015). More details can be found in Section A of the Supplementary Data 1 Text. In some samples, the amount of extracted DNA did not reach 50 ng and was insufficient to establish a sequencing library. Therefore, the extracted DNA was amplified for whole-genome amplification using a REPLI-g UltraFast Mini Kit (Qiagen). By employing Illumina sequencing technology, the complete genomes of 138 field isolates were sequenced. After adapters were trimmed from the reads using Cutadapt 1.15 (Martin 2011), the scaffold of each CnmeGV isolate was assembled by using Ray-2.3.1 (Boisvert et al. 2010) with a k-mer value of 65.

### Comparative analysis of different CnmeGV consensus genomes

Genome annotation of each CnmeGV isolate was performed according to the criteria described previously (Ayres et al. 1994) and corrected by comparison with the reference genome EPDH3 (GenBank accession number OR819345) (Zuo et al. 2024). The whole-genome sequences of the isolates were globally aligned using Mauve (Darling et al. 2010). Multiple sequence alignment was used to identify sequence variations between isolates, including single-nucleotide polymorphisms (SNPs), insertions/deletions (Indels), and nonsynonymous substitutions within open reading frames (ORFs) (Page et al. 2016). Watterson’s estimator (*θ*) of ORFs was determined via default parameters of DnaSP 5.0 (Librado and Rozas 2009).

### SNP-based population structure analysis

To investigate population genetic structure, sequencing reads from each isolate were mapped to the reference genome sequence using BWA-MEM (Li 2013). By using SAMtools for sorting, 138 alignment files were obtained. Variant calling was carried out by employing the bioinformatics pipeline SAMtools mpileup and BCFtools (Li 2011). High-confidence SNPs were filtered by using VCFtools v0.1.15 (Danecek et al. 2011) with the parameters ‘max-alleles 2 -maf 0.05 -remove-indels’. Thus, only biallelic SNPs and loci with a minor allele frequency higher than 5% were retained. Genotype information in groups of individuals was generated with the population programme in Stacks v.2.10 (Rochette et al. 2019).

The CnmeGV population structure was analysed via the discriminant analysis of principal components (DAPC) clustering method. We performed DAPC by using the adegenet package in R 3.5.1 (Jombart 2008) and exploited *a priori* knowledge obtained from sampling. The number of principal components corresponding to the lowest root mean squared error was used as the optimum n.pca in the DAPC (Jombart et al. 2010). The method does not rely on assumptions of Hardy–Weinberg equilibrium and panmixia (Jombart et al. 2010). The DAPC results were visualized via the function scatterplot. The find.cluster function was used to identify the most likely grouping in the data on the basis of the Bayesian information criterion (BIC) calculated for 1 to 40 clusters (Jombart et al. 2010). When assessing *de novo* population structure with DAPC, the optimal number of genetic groups (K) is often determined as that with the lowest BIC values from find.clusters (Jombart et al. 2010).

The BIC analysis indicated that the lowest BIC value occurred at *K* = 4, forming a clear ‘elbow’ pattern, which suggests that the optimal number of genetic groups for CnmeGV isolates from the 4 years is 4.

### Nucleotide diversity calculation

Variant calling was performed by using VarScan version 2.3.9 (Koboldt et al. 2012) for each isolate (detailed in Section C of Supplementary Data 1 Text). To quantify pathogen diversity, the average nucleotide diversity *π*, a standard population genetic statistic, was utilized (Nei and Li 1979). More details can be found in Section C of Supplementary Data 1 Text.

### Observation of the behaviour of first-instar RLF larvae

Since first-instar RLF larvae are too small to be monitored in paddy fields, their habits were monitored in potted rice. Their habits were investigated in a greenhouse maintained at 26°C and 85% humidity with a 16:8 h (light:dark) photoperiod. We designed two experiments to determine which part of the newly hatched rice larvae preferred to feed on, with or without folded leaves. For the experiment without folded leaves, we placed newly hatched larvae into the leaves when the rice reached the tillering stage. After 24 h, we counted the number of larvae at different locations in the rice. For the leaf folding experiments, we first allowed fourth-instar larvae to form folded leaves and then gently squeezed the larvae out of the folded leaves while maintaining the shape of the folded leaves. We subsequently placed newly hatched larvae on the leaves. After 24 h, we counted the number of larvae at different locations on the rice plants. Each experiment had three replications. Fisher’s exact test was used for statistical analysis of the data.

### Determination of CnmeGV abundance

To determine the abundance of CnmeGV in rice leaves, folded leaves, rice sheaths, or paddy soils, real-time quantitative polymerase chain reaction (qPCR) was carried out. A layer of virus soil containing up to 10^6^ copies/g of CnmeGV was covered on the soil surface of potted rice plants, and then the pots were placed in an outdoor natural environment to receive natural rainfall, and rice leaf and sheath samples were collected before and after rainfall. The experiment was conducted using an Applied Biosystems StepOne Real-Time PCR instrument. SYBR Green PCR Mastermix and the primers gran-F and gran-R (Supplementary Table S2 in Supplementary Data 1 Text) were used to amplify the target *granulin* gene of CnmeGV. For the rice leaves, folded leaves, or sheaths, the collected samples were dried in an oven at 65°C or in a vacuum concentrator until a constant weight was reached. The paddy soils were dried in an oven at 65°C. Appropriately weighed dry samples were used to extract viral DNA with a Mag-Bind Soil DNA Kit (OMEGA) according to the instructions. The abundance of CnmeGV was statistically analysed by Student’s *t*-test.

## Results

### A long-term endemic CnmeGV population exhibited substantial genetic variation

To investigate the genetic diversity of a natural baculovirus population, we first collected CnmeGV-infected larvae from *C. medinalis* populations in Dahuai town between 2017 and 2021. Using Illumina sequencing technology, the complete genomes of 138 field isolates (here, an isolate refers to the CnmeGV virions obtained from a single infected larva) were sequenced. The consensus genome lengths of different isolates varied in size, ranging from 111 014 bp to 112 648 bp (mean = 111 946 bp, SD = ± 308.4 bp), and the G + C content ranged from 35.0% to 35.2% (A in Supplementary Data 2). A total of 11 to 12 homologous repeat regions (*hrs*) with a variable number of 10-bp palindrome sequences [TTTACGTAAA] were identified in all the isolates (Section B in Supplementary Data 1 Text). The nucleotide sequence of the genome of each isolate shared 97.6% to 99.6% identity with the reference genome EPDH3 (Section B in Supplementary Data 1 Text, A in Supplementary Data 2). The genomes exhibited high collinearity (Section B in Supplementary Data 1 Text), and no gene inversion was observed. The above results revealed that the genome structure of the CnmeGV population in Dahuai is generally stable.

However, owing to some sequence differences within coding regions that lead to variations such as the gain/loss or fragmentation of ORFs, the predicted number of ORFs varied from 118 to 123 among different isolates (A in Supplementary Data 2, Section B in Supplementary Data 1 Text). All the genomes of the 138 CnmeGV isolates encoded a total of 129 putative ORFs, but 116 ORFs were common to all the isolates.

**Figure 2:**
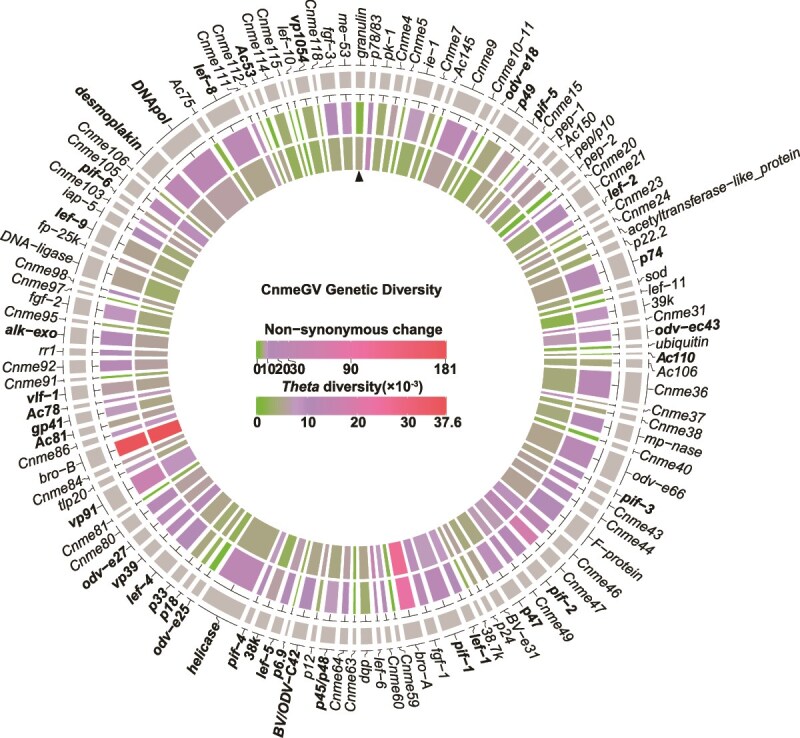
The CnmeGV genes presented genetic diversity. In this circular map, the external elements represent open reading frames (ORFs). The 38 baculovirus core genes are displayed in bold, and the first ORF (granulin) of CnmeGV is indicated by a black triangle. The outer circle is a heatmap depicting the number of nonsynonymous changes per gene. The inner circle shows the genetic diversity measure calculated on the basis of the Watterson estimator (). The values underlying the panels are provided in Section B of the S2 Data.

The genetic diversity of the 116 shared ORFs was calculated via both Watterson’s estimator (*θ*) and the number of nonsynonymous changes (dN). *θ* is equal to the number of segregating sites of a gene divided by a harmonic mean of individuals (Ferretti and Ramos-Onsins 2015). Various factors, such as mutations, gene flow, population structure, genetic drift, and selection pressure, may affect the *θ* value. The *θ* values of the CnmeGV gene sequences within the Dahuai population ranged from 0 × 10^−3^ to 37.6 × 10^−3^ (Fig. 2). The CnmeGV genes with lower *θ* values, such as *pep-2*, *lef-10*, and *Ac145*, may experience strong purifying selection, where harmful mutations are rapidly eliminated, leading to a reduction in genetic variation; in contrast, genes with higher *θ* values, such as *lef-6*, *bro-A*, and *bro-B*, may experience positive selection or balanced selection, where positive selection facilitates the accumulation of favourable mutations for adaptation to the environment, and balanced selection enables the maintenance of multiple alleles in the population.

Nonsynonymous variations can alter the amino acids encoded by genes, potentially affecting protein function. Among the 2883 SNPs identified, at least 1285 nonsynonymous variants were found, and these variants were not evenly distributed throughout the genome (Fig. 2; Section B in Supplementary Data 1 Text). Some genes within CnmeGV, specifically *ac110*, *ac53*, and *odv-e18*, display a reduced quantity of nonsynonymous variation. These genes have relatively more conserved sequences and are likely crucial for virus survival and reproduction. Conversely, certain genes, such as *Cnme47*, *bro-A*, and *bro-B*, presented greater numbers of nonsynonymous variations. These genes might possess more flexible functions in the interactions between the virus and its host or in environmental adaptation. However, we can sort CnmeGV ORFs into the following six functional groups: replication, transcription, *per os* infection, virion assembly and packaging, auxiliary, and functionally unknown ORFs (Zhang et al. 2015). Even though the mean number of nonsynonymous SNPs ranged from 8.08 (virion assembly and packaging) to 26.77 (auxiliary) per kbp, there was no significant difference among the groups (Section B in the Supplementary Data 1 Text). These findings indicate that the Dahuai CnmeGV population has not faced any special selection pressures recently.

**Figure 3:**
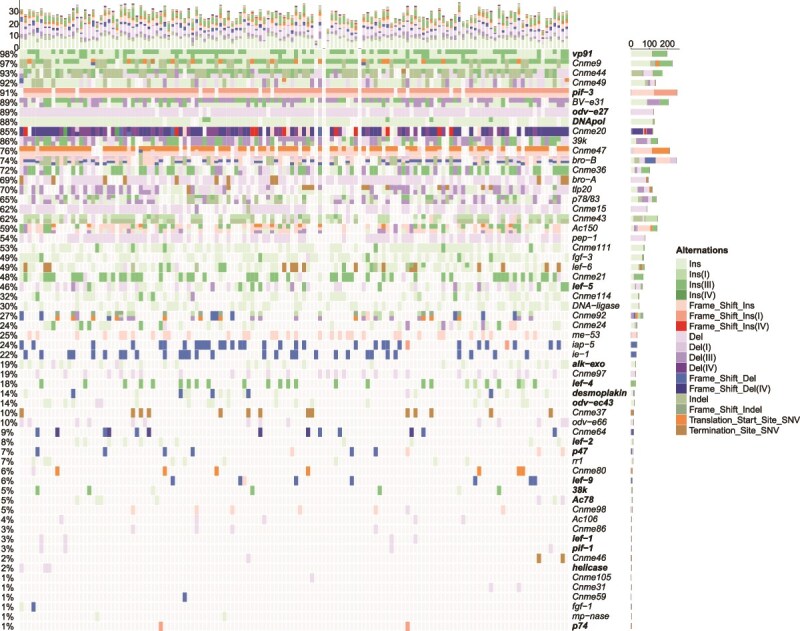
Waterfall plot of SNVs, small Indels, and microsatellite variants causing changes in open reading frame (ORF) length in 138 isolates. Each row represents an ORF, with baculovirus core genes shown in bold. Each column represents an isolate. The different colors in the middle section represent different variant types, as shown on the right. The upper bar graph indicates the number of a certain variant type in an isolate genome, and the right bar graph shows the number of a certain variant type in an ORF and the percentage of variation displayed on the left. Ins, Del, and Indel represent an insertion, a deletion, and an insertion/deletion, respectively, that causes a frameshift mutation. Frame_Shift_Ins, Frame_Shift_Del, and Frame_Shift_Indel represent Ins, Del, and Indel, respectively, that cause frameshift mutations. Ins(I), Ins(III), Ins(IV), Del(I), Del(III), and Del(IV) represent insertions and deletions of single nucleotide repeat (Type I), trinucleotide repeat (Type III) and tetranucleotide repeat (Type IV) microsatellite DNA that do not cause frameshift variations. Frame_Shift_Ins(I), Frame_Shift_Ins(IV), and Frame_Shift_Del(IV) represent insertions and deletions of Type I and IV microsatellite DNA that cause frameshift variations. Translation_Start_Site_SNV and Termination_Site_SNV represent single-nucleotide variants (SNVs) that cause changes in start or stop codons. Frameshift variants involve insertions or deletions that are not multiples of three, and nonframeshift variants refer to insertions or deletions that are multiples of three. When both insertions and deletions exist in an ORF, they are referred to as Indels. The plotted values are provided in Sections A and B in the S3 Data.

Approximately half (56, or 48%) of the shared ORFs exhibited no change in length (Section B in Supplementary Data 2). Among them, 14 ORFs had no nonsynonymous mutations (Section B in Supplementary Data 2). *lef-10* and *pep-2* were the most conserved and did not have any mutations (Section B in Supplementary Data 2). The other half (60, or 52%) of the shared ORFs varied in length in some isolates (Fig. 3, Section B in Supplementary Data 1 Text). ORF lengths may change due to single-nucleotide variations, Indels [including small Indels (1–50 bp) and large Indels (>50 bp)], microsatellite variations, or structural variations (Section B in Supplementary Data 1 Text). Among them, *39k*, *Ac150*, *BV-e31*, *Cnme9*, *Cnme20*, *Cnme47*, *Cnme49*, and *vp91* had more than 10 size variants (Section B in Supplementary Data 1 Text). Proteins with multiple isoforms have diverse functions, allowing them to adjust to changes in host physiology and the ambient environment.

The above results indicate that in a natural baculovirus population capable of sustaining epidemics, genetic variations are highly complex, suggesting that molecular diversity may account for the environmental stability and ongoing effectiveness of the virus.

### The genetic structure of CnmeGV can be distinguished by year, rice-growing season and epidemic wave

**Figure 4:**
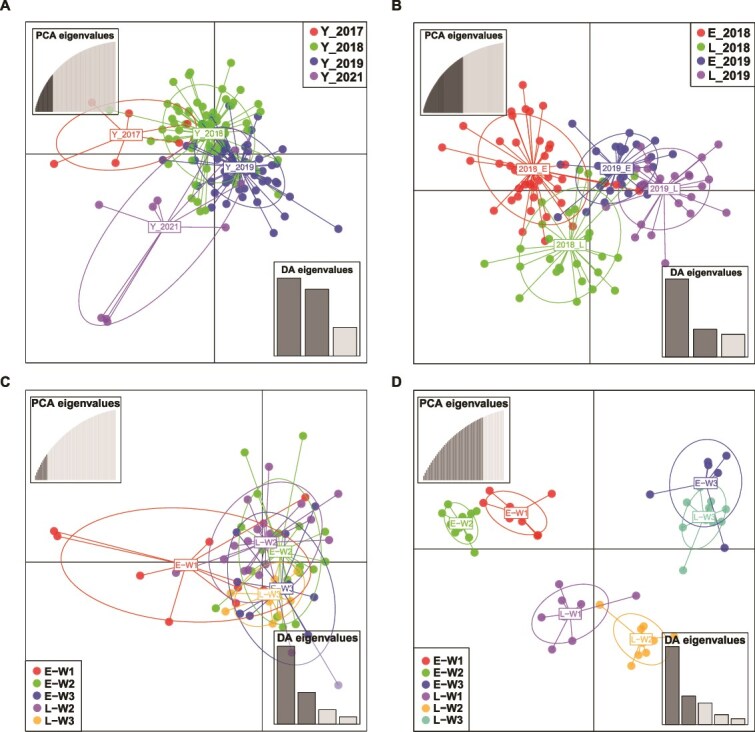
Genetic structure of CnmeGV isolates. Scatterplot obtained via DAPC of CnmeGV isolates: (A) assigned a priori temporal information (by year); (B) from the early and late rice-growing seasons in 2018 and 2019. Plots C and D represent the scatterplots from DAPC of isolates from different CnmeGV epidemic waves in 2018 and 2019, respectively. Individual isolates from the same wave are shown in unique colors. “Y” represents year. “E” and “L” stand for early and late rice, respectively. “W1”, “W2”, and “W3” represent the first wave, the second wave, and the third wave of CnmeGV epidemics, respectively. Individual isolates from the same year (A), the same season (B), or the same wave (C and D) are depicted in unique colors and surrounded by a 95% inertia ellipse. Owing to heavy rain, samples of the W1 of late rice in 2018 were not collected. The W2 samples from both early and late rice in 2018 were composed of isolates collected at two sampling time points.

By evaluating the genetic variation of a pathogen in successive epidemics, it becomes possible to analyse the origin and transmission pathways of the pathogen. Here, DAPC was employed to explore the genetic structure of the 138 CnmeGV field isolates. DAPC is a multivariate method designed to identify and describe clusters of genetically related individuals (Jombart et al. 2010). By mapping the sequencing reads of each isolate to the reference genome (EPDH3), SNPs were extracted, resulting in a dataset containing a total of 1 530 SNPs. Clustering methods revealed population subdivisions, supporting the existence of four genetic groups (Fig. 4a). DAPC groups individuals according to their year of origin. Isolates are correctly assigned to their annual epidemics in an average of 75% of cases, with the probability of correct assignment varying among epidemics from 67% (2017) to 90% (2021). These findings indicated that there were genetic structural differences among the CnmeGV isolates from different years, suggesting that CnmeGV has an annual epidemic cycle.

At the sampling site, a rice cultivation system in which rice is planted and harvested in the same paddy field for two seasons within a year is implemented. In addition, CnmeGV epidemics typically occur in three waves (W1, W2, and W3) during each rice-growing season. Therefore, we conducted a systematic investigation into the prevalence of CnmeGV in 2018 and 2019. DAPC demonstrated that the genetic structures of the CnmeGV isolates could be distinguished according to the rice-growing seasons of early rice and late rice each year (Fig. 4b), suggesting that each rice-growing season corresponds to a CnmeGV transmission cycle. Moreover, the isolates from each rice-growing season could also be distinguished by the epidemic waves of CnmeGV epidemic (Fig. 4c and d), suggesting that different waves of the virus in the CnmeGV transmission cycle in each growing season were linked.

The above results suggest that the genetic structure of CnmeGV can be distinguished by year, rice-growing season and epidemic wave.

### During each rice-growing season, the CnmeGV genomes exhibit higher heterozygosity levels in the initial epidemic wave compared to subsequent waves

To further understand the temporal differentiation of the CnmeGV epidemics elucidated above, we explored the changing patterns of the pathogen within the host. We utilized the average nucleotide diversity π, a standard population genetic statistic employed to summarize diversity (Nei and Li 1979), to quantify pathogen diversity between and within individual hosts. The calculations over all the sites of the consensus genomes indicated that the nucleotide diversity ranged from 0.00012 to 0.0035 (Section B of Supplementary Data 4), with a mean value of 0.00034. As reported previously, most sites are conserved both between and within individual baculovirus-infected hosts (Kennedy and Dwyer 2018). Since conserved sites provide limited information on genetic variation, we identified segregated sites (defined as sites where alternative variants were conserved in more than seven samples [ ~5%]) within samples. Comparisons between consensus sequences identified 928 segregating sites at the between-host level. Nucleotide diversity at the segregating sites was calculated via the consensus sequences of all the isolates, and the results revealed that the between-host nucleotide diversity (π) was 0.309 (Section C of Supplementary Data 4). Within individual isolates, the nucleotide diversity ranged from 0.001 to 0.225 (mean = 0.042, SD = ±0.059, Section D of Supplementary Data 4).

**Figure 5:**
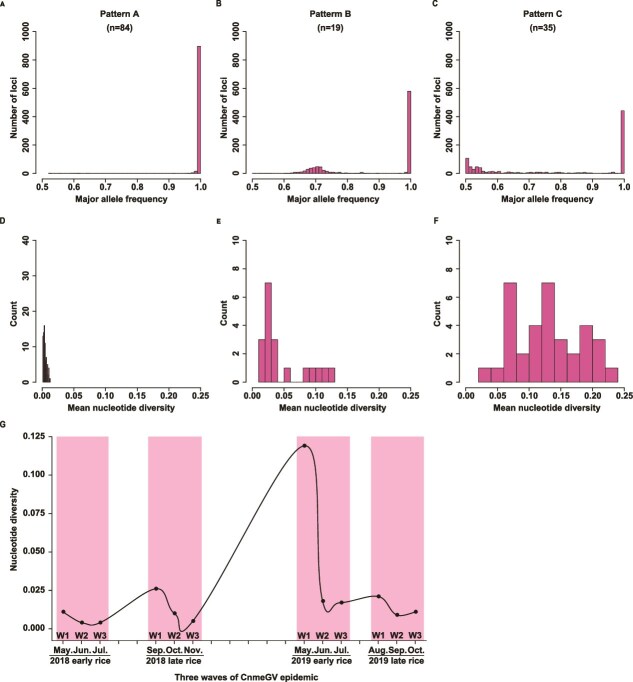
Patterns of nucleotide diversity in pathogens within hosts. Plots A, B, and C represent the three distribution patterns of allele frequencies within a single individual at 928 segregating sites. The number in parentheses is the number of isolates belonging to that specific pattern. A, The absence of diversity in Pattern A implies that the virus population within these isolates consists of only a single virus strain. B, The bimodal distributions in Pattern B suggest that these isolates contain exactly 2 virus strains. C, The high diversity yet lack of bimodality in Pattern C indicates that these isolates consist of more than 2 virus strains. Plots D, E, and F show the distributions of the mean nucleotide diversity of individual isolates belonging to Patterns A, B, and C, respectively. G, The CnmeGV genomes exhibit higher heterozygosity levels in the initial epidemic wave compared to subsequent waves during each rice-growing season. The x-axis indicates the months from May 2018 to Oct 2019, and the y-axis represents the median of the estimated nucleotide diversity. Owing to the scarcity of RLF caused by heavy rains at the beginning of tillering in 2018, we were unable to obtain the median nucleotide diversity of the first wave of CnmeGV isolates in this season, so this value was taken as the average of the values from the other three growing seasons.

Analysis of the variation at the segregating sites of the CnmeGV genomes within each isolate revealed that these sites were polymorphic in some isolates but not in others (Section C in Supplementary Data 1 Text), suggesting that some infections likely involved multiple viral genotypes, while others appeared to be dominated by a single detectable genotype at the time of sampling. Three patterns of nucleotide diversity could be distinguished. Pattern A showed a single distribution of allele frequencies (Fig. 5a), indicating a lack of diversity in the virus population within these isolates and the presence of only one strain of one isolate. There were 84 isolates with viral genetic structures that belonged to Pattern A, with nucleotide diversity ranging from 0.001 to 0.013 (Fig. 5d, Section C of Supplementary Data 1 Text). Pattern B showed bimodal distributions of allele frequencies (Fig. 5b), indicating that exactly two virus strains were present in each isolate. The viral populations in 19 isolates belonged to Pattern B, with nucleotide diversity ranging from 0.011 to 0.129 (Fig. 5e, Section C of Supplementary Data 1 Text). Pattern C showed high diversity but a lack of bimodality (Fig. 5c), suggesting that each isolate consisted of more than 2 virus strains. The viral populations of 35 isolates belonged to Pattern C, with nucleotide diversity ranging from 0.033 to 0.225 (Fig. 5f, Section C of Supplementary Data 1 Text). We further discovered that the ratio of mixed infection (Patterns B and C) to single infection (Pattern A) was negatively correlated with the number of waves of CnmeGV epidemic (Section C of Supplementary Data 1 Text).

Overall, the above results indicate the CnmeGV genomes exhibit higher heterozygosity levels in the initial epidemic wave compared to subsequent waves (Fig. 5g). Notably, the W1 population in the early rice season of 2019 showed an unusually high level of nucleotide diversity. The underlying cause of this spike remains unclear. It is possible that multiple viral genotypes co-circulated or co-infected the host population during that period, potentially influenced by transient ecological or environmental conditions. Therefore, this argument is somewhat speculative. To determine whether this observation reflects a broader trend or a stochastic anomaly, continuous and broader sampling across multiple years, combined with additional replicates, will be necessary.

### Neonatal RLF larvae may be infected with CnmeGV in leaf sheaths or old folded leaves of rice

The decreasing trend in the average nucleotide diversity of virus genomes during a seasonal CnmeGV epidemic suggested that bottleneck effects may occur in the transmission of CnmeGV populations (Kennedy and Dwyer 2018). On the one hand, although the pathogen population in the environment may be large, new infections are triggered by a small number of pathogens, which typically leads to bottlenecks. On the other hand, the susceptibility to infection tends to decrease with increasing larval age in many baculovirus–host systems (Federici 1986, Teakle et al. 1986, Kirkpatrick et al. 1998, Cory and Myers 2003). Moreover, some insects can be infected when exposed to lower doses of virus particles (OBs), and even in extreme cases, a single OB may have a fatal effect on newly hatched larvae (Fuxa 1987, Fuxa et al. 1988, Sun et al. 2006). Therefore, observing the living habits of young larvae, especially first-instar larvae, is crucial to understanding outbreaks of baculoviruses.

**Figure 6:**
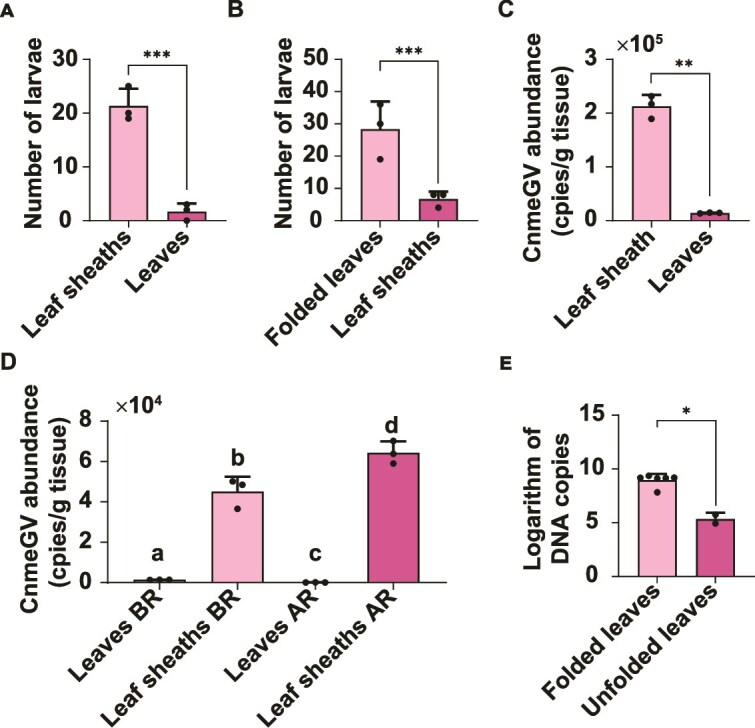
Observation of the aggregation of neonatal larvae and determination of the abundance of CnmeGV in rice. A, In the absence of folded leaves, there were significantly more newly hatched RLF larvae foraging in the leaf sheaths than in the leaves (Fisher’s exact test, ***P = 3.80 × 10^−14^). B, There were significantly greater numbers of newly hatched RLF larvae foraging on the folded leaves than on the leaf sheaths (Fisher’s exact test, ***p = 7.07 × 10^−11^). C, Abundance of CnmeGV on the surface of leaves and leaf sheaths of rice in a natural paddy field (Student’s t test, **p = 4.84 × 10−7). D, Abundance of CnmeGV on the surface of the leaves and leaf sheaths of potted rice before and after rainfall. “BR” indicates before rainfall, and “AR” indicates after rainfall (Student’s t test; the same letters indicate no significant difference, *p* > 0.05). E, Abundance of CnmeGV on folded leaves and unfolded leaves in the paddy field of Dahuai town (Student’s t test, **p = 0.006). Real-time quantitative polymerase chain reaction (qPCR) was used to determine the abundance of CnmeGV.

Female adults usually lay eggs near the tips of leaves, sometimes on leaf sheaths. There are few reports on the living habits of first-instar RLF larvae, possibly because they are too small to be easily observed in the field. In this study, we experimentally confirmed that after hatching, most W1 neonatal larvae entered the leaf sheaths (Fig. 6a), whereas W2 and W3 neonatal larvae mostly entered folded leaves (Fig. 6b) and began feeding. Through real-time quantitative polymerase chain reaction (qPCR), CnmeGV was found to be enriched in the leaf sheath of rice at the tillering stage because of rainfall at the sampling site (Fig. 6c) or in the laboratory (Fig. 6d). At the sampling site, the amount of CnmeGV in the old folded leaves was much greater than that in the unfolded leaves (Fig. 6e). These observations indicate that the RLF W1 larvae are infected with mainly CnmeGV in the leaf sheaths, whereas the RLF W2 and W3 larvae are infected in the old folded leaves.

## Discussion

Owing to the scarcity of individuals with overt disease and the sporadic nature of the epidemic, our knowledge of baculovirus ecology is very limited, which impacts the effective utilization of these viruses in biocontrol (Cory et al. 1997, Sun et al. 2006, Myers and Cory 2016, Williams 2018). To further understand the ecology and evolution of host–pathogen interactions and to develop more effective biocontrol strategies, we present a dynamic pattern of changes in the genetic structure of a natural baculovirus population.

By sequencing viral genomes extracted from 138 CnmeGV-infected RLF larvae collected over several years from the same paddy field, we revealed substantial genetic variation within the CnmeGV population, reflecting both differences between hosts and single- or mixed-genotype infections within individual hosts. By using DAPC and pairwise fixation index (Fst), we found that the population genetic structure of 392 CnmeGV isolates collected from 12 sampling sites in Guangdong and Guangxi showed geographical differences (unpublished data). This spatial divergence suggests restricted gene flow among virus populations in different regions and implies that long-distance migration of *C. medinalis* may play a limited role in shaping the viral population structure. Additionally, the present study revealed that the genetic structure of CnmeGV isolates collected from the DH population could be differentiated by year. The coexistence of both spatial and temporal structuring supports the argument that the CnmeGV epidemics are likely to be caused annually by a local virus reservoir rather than by new exogenous strains or latent infection of the RLF. If long-distance migration of the host *C. medinalis* played a major role in viral dissemination, we would expect to observe a homogenization of viral genetic structure across regions. We acknowledge that further multi-year sampling and broader geographic coverage are needed to strengthen this conclusion, but our current data provide preliminary evidence favouring localized epidemic origins. Studies have indicated that soil is an important reservoir for baculovirus OBs. These OBs can survive in soil for decades and are crucial for the continuous occurrence and spread of baculovirus epizootics in natural insect populations (Thompson et al. 1981, Cory and Myers 2003, Williams 2023). Fuxa and Geaghan (Fuxa and Geaghan 1983) reported that the most significant factor affecting viral prevalence in the fall armyworm *Spodoptera frugiperda* populations was overwintering baculovirus present in the soil. The amount of CnmeGV in the Dahuai town paddy field was 1.7 × 10^7^ copies/g soil, which was approximately 4576 times greater than that in a paddy field with no CnmeGV epidemic (Part E of Supplementary Data 1 Text), indicating that the soil is a reservoir of CnmeGV at the sampling sites.

OBs in soil can be redistributed to plants through rain splash and soil disturbance, and the growth of seedlings (Olofsson 1988, Fuxa et al. 2001, Fuxa 2008) and their translocation may be greater in shorter, less dense crop types (Cory et al. 1997). After rice seedlings are transplanted, the CnmeGV OBs in the soil may be distributed on rice leaves. Here, we demonstrated that rainfall enriches CnmeGV in the leaf sheath of rice (Fig. 6c and d), which may be due to rainwater depositing CnmeGV OBs from the leaves into the leaf sheaths. Since direct exposure of baculoviruses to sunlight can reduce or even inactivate their infectivity (Podgwaite and Mazzone 1981, Biever and Hostetter 1985), the sheath-like structure of the leaf sheath can block sunlight; thus, the OBs in leaf sheaths are protected from sunlight and remain viable for longer periods. Generally, three CnmeGV epidemics can occur during the rice-growing season at our sampling site. Considering that the nucleotide diversity index of the W1 isolates was lower than that in the soil (Part D of Supplementary Data 1 Text) and that the genetic structure of all the sampled isolates could be distinguished by year, these findings suggest that the annual starting virus strains constitute only a random subset of the total viral strains from this soil reservoir.

**Figure 7:**
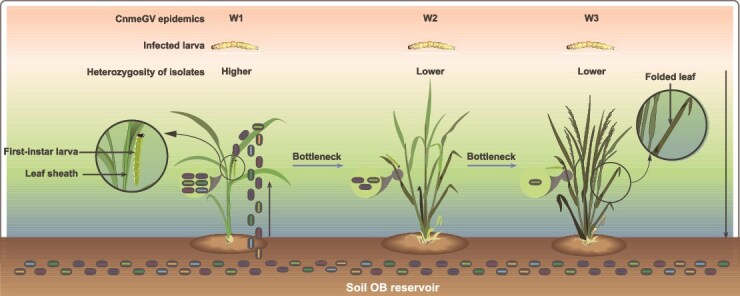
A potential virus transmission route during the CnmeGV epidemics across three waves in the same rice-growing season. In the ecological cycle of the CnmeGV, OBs in the soil are transported back onto rice leaves perhaps through rain splash, soil disturbance, etc. Then rainfall washes OBs into the leaf sheaths, where they accumulate. The first-instar larvae of RLF feed on leaf sheaths that are contaminated with OBs and thereby become infected with the virus. When first-instar larvae grow to the second instar and beyond, they move to the middle of the leaves and roll up the leaves longitudinally to form folded leaves. OBs enter the old folded leaves through the feces of infected larvae. The first-instar RLF larvae belonging to the second and third waves become infected with the virus by feeding on leaves contaminated by OBs in feces. As the CnmeGV epidemic progresses, the average nucleotide diversity of CnmeGV isolates tends to decrease, and the bottleneck effects may occur in the transmission of the CnmeGV populations.

Given the similar genetic structures observed among CnmeGV isolates within the same rice-growing season (Fig. 4b), we propose that a potential transmission route may have existed among the three waves of CnmeGV epidemics (Fig. 7). However, additional long-term sampling and further analysis are required to confirm this speculation. The first-instar larvae of RLF tend to nibble on mesophyll tissue in the leaf sheath (Fig. 6a). Baculovirus infection can be established in first-instar larvae at very low viral doses (Mitchell et al. 1985, Kunimi et al. 1997). Therefore, the enrichment of both CnmeGV and first-instar RLF larvae in leaf sheaths increases the probability of ingestion of infectious viral particles by susceptible hosts. When first-instar larvae grow to the second instar and beyond, they move to the middle of the leaves and roll up the leaves longitudinally to form folded leaves. Interestingly, our study revealed that once the leaves were folded, most of the newly hatched first-instar larvae moved in and fed inside the fold (Fig. 6b). This behaviour may be due to the larvae’s ability to sense the odour cues left in the rolled leaves by the previous batch of larvae, thus choosing to enter that area. Previous studies have shown that OB enters the environment through the faeces of infected larvae as one of the routes of baculovirus transmission (Vasconcelos 1996). We demonstrated that CnmeGV was present at very high concentrations in folded leaves in areas with high CnmeGV endemicity (Fig. 6e), suggesting that infective OBs may be continuously shed via faeces by the CnmeGV-infected RLF. Notably, folded leaves also protect CnmeGV OBs from inactivation by UV light. Thus, for first-instar RLF larvae belonging to the second and third waves, the folded leaves are the sites where they become infected when they feed on leaves contaminated with CnmeGV. As the CnmeGV epidemic progressed, the average nucleotide diversity of the CnmeGV isolates tended to decrease (Fig. 5g), suggesting that bottleneck effects occurred in the transmission of the CnmeGV populations. Bottleneck effects are common during intrahost replication and interhost transmission of pathogens. In the W1 stage, the virus population experienced a significant bottleneck effect, mainly due to the combined effects of the founder effect and replication drift (replication drift leads to random amplification of some genotypes and random loss of other genotypes, thereby limiting overall diversity), resulting in a significant decrease in the genetic diversity of the virus population in subsequent waves (W2 and W3). In the initial infection period (W1), only a very small number of virus particles successfully infect host individuals, triggering a strong genetic bottleneck and significantly reducing the genetic diversity of the virus population. In addition, the viral particles excreted in the faeces of infected larvae may further aggravate the genetic bottleneck effect. Since faeces contain only a limited number of viral genotypes and only some particles can be ingested by subsequent larvae, this may have narrowed the genetic basis of the virus. In the W2 and W3 stages, newborn larvae are exposed to viral infection by feeding on leaves that are heavily contaminated by the faeces of infected larvae. During this process, the viral replication drift effect continues to occur, exacerbating genetic drift, resulting in low levels of genetic diversity in CnmeGV isolates in the W2 and W3 stages. Previous studies have demonstrated that bottlenecks and replicative drift appear to be the main drivers of diversity at sites that segregate at the population level of the gypsy moth baculovirus (Kennedy and Dwyer 2018). High levels of genetic variation in a baculovirus population may lead to successful infection of hosts with varying degrees of disease resistance, enabling the population to perpetuate (Elderd et al. 2008).

Our study reveals a potential route of viral transmission for CnmeGV epizootics in natural environments: The first wave (W1) of epidemics was caused by the first-generation first-instar larvae of RLF feeding on viral OBs enriched in rice leaf sheaths; while the second and subsequent epidemics (W2+) were mainly caused by the new first-instar larvae feeding on virus-contaminated folded leaves excreted by the previous generation of infected larvae (Fig. 7). In other words, the W1 epidemic occurred in the leaf sheaths, and the W2+ epidemic was concentrated in the folded leaves. This route may have been formed by the long-term evolution of CnmeGV and led to a gradual increase in host prevalence (Section E in Supplementary Data 1 Text). This study provides new ideas and methods for the use of biological pesticides to control pests and helps formulate more effective biological control strategies. We suggest the precise spraying of CnmeGV into the leaf sheath of rice before the emergence of first-generation larvae in each rice-growing season. Spraying CnmeGV only once during the rice-growing season can effectively allow the virus to exert control effects, thereby reducing the amount of the virus used. As a potential biological pesticide, CnmeGV can reduce the reliance on chemical pesticides, lower environmental pollution, and promote the sustainable development of agriculture.

Since the research was only conducted in Dahuai town, where the long-term prevalence of CnmeGV was discovered, a notable limitation is the lack of comprehensive data from multiple regions. However, this research fills a gap in the lack of long-term field data on baculoviruses and can provide a basis and reference for future research on the genetic structure of viruses in different regions.

## Author contributions

B.C., Y.Z., and K.Y. conceptualized the idea. B.C. performed the analysis and data visualization. B.C., Y.Z., Y.L., H.Z., J.Y., M.Y., and K.Y. analysed the epidemiological data. B.C., H.Z., Y.G., and J.Y. collected data and completed the sequencing and assembly of the viral genome. B.C. wrote the original draft of the paper with input from K.Y., J.Y., and B.C. created a transmission map of the virus. All authors have read and agreed to the published version of the paper.

## Supplementary Material

S1_Text-VEVOLU-2025-014_R2_veaf055

Supplementary_Data_1_veaf055

Supplementary_Data_2_veaf055

Supplementary_Data_3_veaf055

Supplementary_Data_4_veaf055

Supplementary_Data_5_veaf055

## Data Availability

The raw sequence data reported in this article have been uploaded to the NCBI nr database. The accession numbers are PQ824721-PQ824789 and PQ824790-PQ824858.
