# Supplementary material for: Revealing the potential transmission route of Cnaphalocrocis medinalis granulovirus capable of persistently causing granulosis epidemics
Source: Virus Evol. 2025 Jul 25;11(1):veaf055. doi: 10.1093/ve/veaf055 (PMC12371407; doi:10.1093/ve/veaf055)
Supplement: S1_Text-VEVOLU-2025-014_R2_veaf055 [file s1_text-vevolu-2025-014_r2_veaf055.docx]

**Supplemental Information**

# A Collection of isolates, sequencing, and bioinformatics analysis

Cnaphalocrocis medinalis granulovirus (CnmeGV) was first discovered in Fiji and subsequently in India ([Jacob et al., 1973](#_ENREF_23); [Steinhaus and Marsh, 1962](#_ENREF_48)). In China, CnmeGV was initially discovered and isolated in Enping County, Guangdong Province ([Pang et al., 1981](#_ENREF_44)). Notably, in Dahuai town of Enping County, CnmeGV can cause granulosis in *Cnaphalocrocis medinalis* for a prolonged period ([Zhang et al., 2014](#_ENREF_53); [Zuo et al., 2024](#_ENREF_56)).

The occlusion bodies (OBs) of CnmeGV were purified from infected larvae following the protocol described elsewhere ([O'Reilly et al., 1992](#_ENREF_43)). Briefly, the milky white larvae were individually crushed and homogenized to release the OBs. The pieces of cadavers were removed by filtering each virus solution through four layers of gauze into a 1.5-ml centrifuge tubes. For purifying the OBs, differential centrifugation was carried out as follows: the filtrate was centrifuged at 20 × g for 5 minutes, and then the supernatant was centrifuged at 10,000 × g for 5 minutes. Each pellet was re-suspended in 1 ml of 0.1% SDS solution, and the differential centrifugation was repeated 2 to 3 times. Next, each pellet was re-suspended in 1 ml of distilled water, and the differential centrifugation was repeated 2 to 3 times. Finally, the pellet was re-suspended in 120 µl of distilled water.

Viral DNA was extracted as described previously ([O'Reilly et al., 1992](#_ENREF_43); [Zhang et al., 2015](#_ENREF_54)). Briefly, to release virions from OBs, 60 µl of DAS buffer (0.1 M Na_2_CO_3_, 0.17 M NaCl, 0.01 M EDTA, pH 10.5) was added to each virus solution and incubated at 37 °C for 30 minutes. Then, 1% 20 mg/ml proteinase K was added and incubated overnight at 37 °C. Subsequently, 10% SDS (so that the final concentration of SDS was 1%) was added. Each tube was rotated at low speed on a rotary shaker for 10 minutes and then incubated for 30 minutes at 50 °C to release DNA from the virions.

Genomic DNA was extracted from the lysates by using a viral DNA&RNA extraction Kit (GenStar, USA). The quantity of DNA was determined using a Qubit^®^ 4.0 Fluorometer (Thermo Fisher Scientific).

For each of the CnmeGV isolates, a next-generation sequencing library was established using the VAHTS Universal Pro DNA Library Prep Kit for Illumina. Briefly, the prepared viral DNA was fragmented with transposase, and adapters containing an 8-base pair barcode and sequencing primer binding sequences were ligated at the ends of the fragments ([Zhang et al., 2022](#_ENREF_52)). The products were sorted by magnetic beads, and fragments of approximately 400 base pairs were enriched. The Illumina sequencing technology was employed to sequence the genomes of CnmeGV isolates, generating an average of 6,056,029 reads for each isolate (Section A of S1 Data). Owing to the large data size of these reads, assembling the consensus sequence of CnmeGV isolates is time-consuming. To expedite genome assembly, the Seqtk tool was utilized to extract 2 to 5 million reads from a dataset that had used Cutadapt 1.15 ([Martin, 2011](#_ENREF_40)) to remove the adapters in all reads. Subsequently, scaffolds of CnmeGV isolates were assembled by using Ray-2.3.1 ([Boisvert et al., 2010](#_ENREF_4)) with a k-mer value of 65. The longest scaffold was then further refined using Geneious 10.1.2 ([Kearse et al., 2012](#_ENREF_25)) to obtain the consensus sequence for each isolate. The overall mean genome coverage was 9,969 × per sample, with a range between samples of 634 × to 17,461 × (Fig. S1).


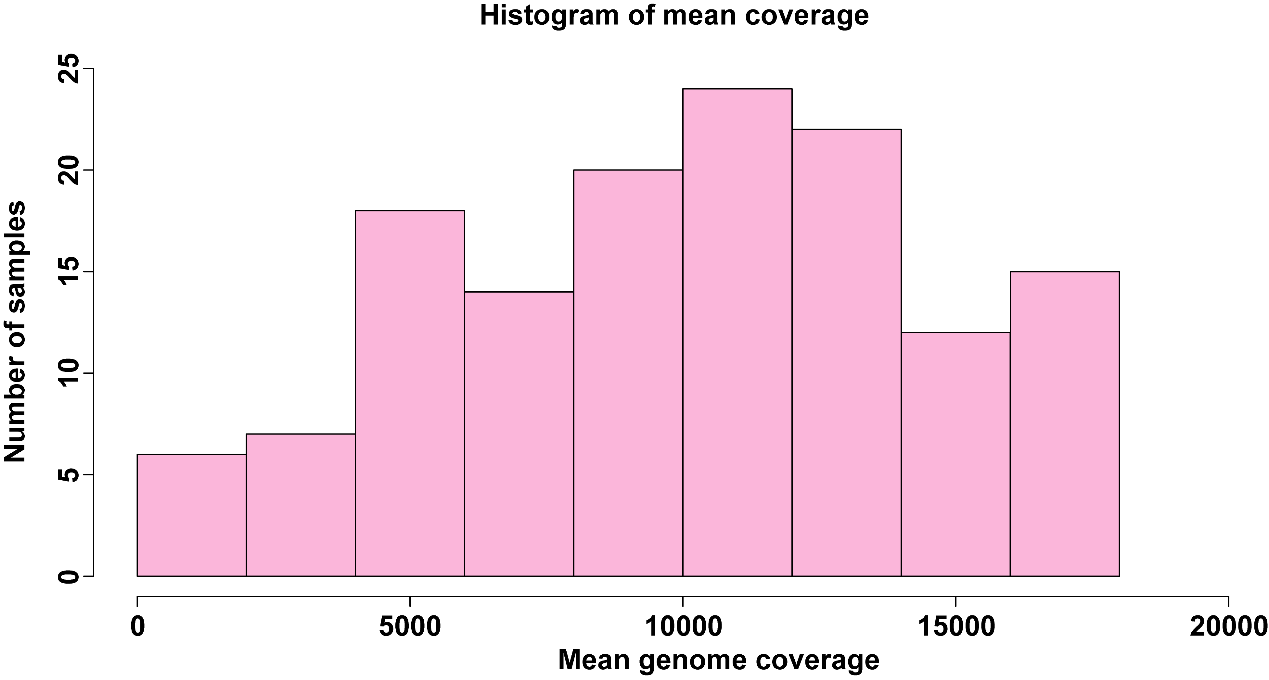


**Fig. S1:** Mean genome coverage for each of the 138 sequenced samples. The values underlying the histogram are provided in Section A of S1 Data.

# B Comparative analysis of different CnmeGV consensus genomes

The CnmeGV genome annotation was preformed according to the criteria described previously ([Ayres et al., 1994](#_ENREF_1)). Open reading frames (ORFs) were predicted by using Geneious 10.1.2 and homologous repeated regions (*hrs*) were identified by using Tandem Repeat Finder ([Benson, 1999](#_ENREF_2)). Both ORFs and *hrs* were corrected by comparison with the annotation of the reference genome EPDH3 (GenBank accession number OR819345) ([Zuo et al., 2024](#_ENREF_56)).

Although the gene order or orientation of baculovirus genomes in different species may vary ([Herniou et al., 2003](#_ENREF_20); [Huang et al., 2019](#_ENREF_22)), the baculovirus genomes of the same species are generally colinear ([Harrison et al., 2016](#_ENREF_19); [Wennmann et al., 2017](#_ENREF_49)). By using Mauve ([Darling et al., 2010](#_ENREF_9)), global alignment of the whole genome sequences of several viral isolates showed that the genomes of CnmeGV exhibited high collinearity and no gene inversion (Fig. S2). The nucleotide sequences of the *hr4*, *bro-A*, and *bro-B* genes and their surrounding regions were less conserved than other regions of the genomes (Fig. S2).

*Hr* is one of the characteristics found in most baculovirus genomes and was first identified in Autographa californica multiple nucleopolyhedrovirus (AcMNPV) ([Cochran and Faulkner, 1983](#_ENREF_7); [Pearson et al., 1992](#_ENREF_45)). *Hrs* may serve as DNA replication origins or transcription enhancers during viral replication ([Habib and Hasnain, 1997](#_ENREF_16); [Pearson and Rohrmann, 1995](#_ENREF_46)). The center of CnmeGV *hrs* is an identical perfect 10-bp palindrome sequence [TTTACGTAAA]. For the 138 isolates analyzed in this study, 82 isolates had 11 *hrs*, which were the same number and distribution as the reference isolate EPDH3. However, there were 56 isolates with an extra *hr*, which consisted of a single palindromic sequence and was named *hr8a* (Fig. S3). These *hrs* are scattered along the genome in intergenic regions, and the number of palindromic sequences varies between isolates (Fig. S3, Section A of S2 Data). The size changes of 9 *hrs* might be associated with the gain or loss of the palindromic sequences. *Hr4* was the longest *hr* in CnmeGV and showed the greatest variation, with 16 variants (Fig. S3). The size variations observed within *hrs* might be the result of slipped-strand mispairing during DNA replication ([Bzymek and Lovett, 2001](#_ENREF_5); [Levinson and Gutman, 1987](#_ENREF_33)).

By comparing the gene content of the 138 CnmeGV consensus genomes, a total of 129 putative ORFs were found in these isolates, and 116 of them were shared by all genomes. There are 13 ORFs that are not common to all isolates, including *Cnme10*, *Cnme11*, *Cnme10-11*, *Cnme58*, *Cnme79*, *Cnme3a*, *Cnme26a*, *Cnme27a*, *Cnme57a*, *Cnme84a*, *Cnme85a*, *Cnme91a*, and *Cnme97a* (Section A of S2 Data). Among them, some ORFs showed a high frequency of occurrence, including *Cnme10-11* (136/138, 98.6%), *Cnme79* (130/138, 94.2%), and *Cnme84a* (102/138, 73.9%) (Section A of S2 Data). Due to insertions/deletions (Indels) or substitution in the genomes of some isolates, five new putative ORFs (*Cnme3a*, *Cnme26a*, *Cnme27a*, *Cnme57a*, and *Cnme84a*) were observed through the alignment of the MAFFT program of Geneious software (Section C of S2 Data). With the exception of *Cnme84a*, which is significantly homologous to *bro-B*, the amino acid sequences of these new hypothetical proteins have no significant identity with any other sequences in GenBank. *Cnme84a* is located upstream of *bro-B*. The *bro* gene might be the result of viral genome recombination. Given that viral genome recombination allows gene duplication and loss ([Bideshi et al., 2003](#_ENREF_3); [De Jong et al., 2005](#_ENREF_10)), *Cnme84a* may be obtained through *bro-B* replication or *bro-B-like* sequence recombination. Duplication and loss of *bro* genes seem to be common in baculovirus genotypic variation ([Harrison et al., 2012](#_ENREF_18); [Zhou et al., 2012](#_ENREF_55)). The gain or loss of ORFs in isolates may affect the phenotype of the virus. For instance, the presence or absence of the *bro* gene may be involved in influencing the toxicity of MacoNPV-A viruses v90/4 and v90/2 ([Li et al., 2005](#_ENREF_37)).

Single nucleotide polymorphisms (SNPs), Indels, and nonsynonymous substitutions were extracted from 116 shared ORFs. Fig. S4 shows that protein size is weakly correlated with the number of nonsynonymous changes. The auxiliary genes *bro-B* and *bro-A* presented the highest level of nonsynonymous nucleotide changes, followed by *Cnme47* and the baculovirus core gene *vp91* (Fig. S4). The BRO protein of Bombyx mori nucleopolyhedrovirus has been shown to be a nucleoplasmic shuttle protein that utilizes the CRM1-mediated nuclear export pathway ([Kang et al., 2006](#_ENREF_24)). *vp91* encodes an essential component of the *per os* infection complex, which binds to insect midgut cells and is involved in primary infection ([Peng et al., 2010](#_ENREF_47)). Fig. S5 shows that the distribution of nonsynonymous changes in 116 shared ORFs in six ORF functional categories. Analysis of variance was used for comparison between groups (*p* > 0.05). Notably, 876 nonsynonymous substitutions were associated with changes in amino acid polarity (Section B in S2 Data), which are more likely to alter protein function ([Hanada et al., 2006](#_ENREF_17)).

Sixty ORFs varied in length due to single nucleotide variations (SNVs), Indels (including small Indels [1–50 bp] and large Indels [> 50 bp], microsatellite variations, or structural variations (S3 Data). Eight ORFs had more than 10 size variants (Section B of S3 Data). SNVs may alter the position of the stop codon or the first methionine of ORFs. The ORFs with the stop codon position change included *bro-A*, *Cnme37*, *Cnme44*, *Cnme46*, *lef-6*, and *tlp20* (Fig. 3 in the main text, Section A and B in S3 Data). The ORFs with the first methionine change included *Ac150*, *Cnme9*, *Cnme47*, *Cnme49*, *Cnme80*, and *Cnme92* (Fig. 3 in the main text, Section A and B in S3 Data).

Small Indels were present in 59 ORFs except *Cnme37* (Fig. 3 in the main text, Section A and B in S3 Data). Here, *ie-1* was shortened or extended by tens or hundreds of nucleotides in 30 CnmeGV isolates (21.7%) due to the insertion or deletion of a single nucleotide. IE-1 can transactivate the early genes of AcMNPV ([Guarino and Summers, 1986](#_ENREF_14)), may be involved in the negative regulation of certain genes ([Leisy et al., 1997](#_ENREF_32)), and is essential for transient DNA replication ([Kool et al., 1994](#_ENREF_29); [Lu and Miller, 1995](#_ENREF_39)). The changes of *ie-1* may affect its function in regulating viral replication. There were three ORFs with large Indels, including *bro-A*, *Cnme20*, and *Cnme24* (Section C of S3 Data). Viral genomes with large deletions have been proven to bring about co-adaptive effects for some baculovirus populations ([López-Ferber et al., 2003](#_ENREF_38)).

Microsatellite DNA is a type of short tandem repeats (STRs) consisting of 1 to 6 nucleotides in tandem. In the ORFs of CnmeGV, single nucleotide repeat (Type I) microsatellite DNA, trinucleotide repeat (Type III) microsatellite DNA, and tetranucleotide repeat (Type IV) microsatellite DNA were detected. Among them, there were 8 ORFs with Type I microsatellite DNA changes, namely *Cnme43*, *Cnme49*, *Cnme92*, *Cnme114*, *iap-5*, *lef-4*, *p74*, and *pif-3* (Fig. 3 in the main text, Section A and B in S3 Data). There were 23 ORFs with Type III microsatellite DNA changes, including *38k*, *39k*, *Ac150*, *BV-e31*, *Cnme9*, *Cnme15*, *Cnme21*, *Cnme24*, *Cnme36*, *Cnme43*, *Cnme44*, *Cnme49*, *Cnme92*, *Cnme111*, *Cnme114*, *DNApol*, *fgf-3*, *lef-4*, *lef-5*, *lef-6*, *p78/83*, *tlp20*, and *vp91* (Fig. 3 in the main text, Section A and B in S3 Data). Only *Cnme20* had Type IV microsatellite DNA changes (Fig. 3 in the main text, Section A and B in S3 Data). Microsatellite DNA changes may have diverse phenotypic consequences. In Cydia pomonella granulovirus (CpGV), a Type IV microsatellite DNA change in a transcriptional activator (PE38) has been demonstrated to be a crucial target for the codling moth’s type I resistance to CpGV-M ([Fan et al., 2020](#_ENREF_12); [Gebhardt et al., 2014](#_ENREF_13)).

Twenty-three ORFs showed frame-shift variations in certain isolates, primarily due to nucleotide insertion or deletion (Fig. 3 in the main text, Section A and B in S3 Data). Frame-shift variation of baculovirus ORF may have significant effects on protein structure, function, viral fitness and evolution.

There were 8 ORFs that were split or fused due to Indels, including *bro-A*, *bro-B*, *Cnme9*, *Cnme10-11*, *Cnme46*, *Cnme47*, *DNA-ligase*, and *p78/83* (Fig. S6, Section D of S3 Data). Fusion or fission of adjacent genes may be a common mechanism for baculovirus evolution. For instance, at least four pairs of adjacent ORFs fusions were found in AcMNPV ([Chen et al., 2013](#_ENREF_6)). Just like EPDH3, the tandem ORFs *Cnme10* and *Cnme11* were fused to produce *Cnme10-11* in 98.6% (136 isolates) of isolates (Section A in S2 Data), indicating that *Cnme10-11* is ubiquitous in the CnmeGV Dahuai population and may provide an adaptive advantage. The fission and fusion of *Cnme10* and *Cnme11* led to completely unique amino acid sequences of the ORFs. A BLASTp comparison showed that the amino acids of *Cnme10-11* have very high homology with thymidylate kinase (BLASTP E value < 1e-05), which is a key enzyme for pyrimidine synthesis ([Cui et al., 2013](#_ENREF_8)).


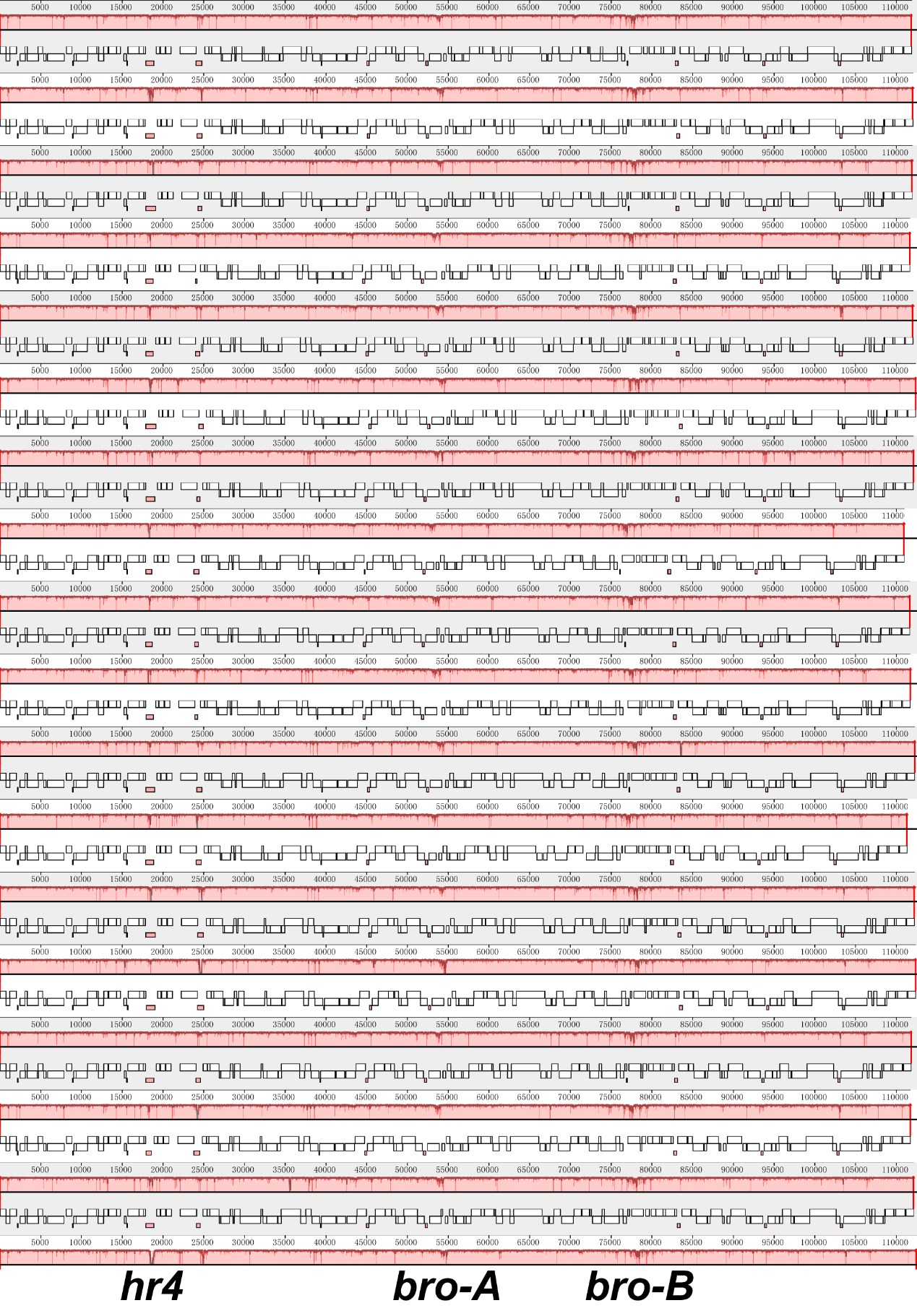


**Fig. S2:** Eighteen CnmeGV genome sequences were aligned using Mauve. The *hr4* is the longest and most structurally complex *hr* in the CnmeGV genome. The correction of *hr4* in these isolates was completed by *Sanger* sequencing. Block outlines of the same color correspond to Locally Collinear Blocks (LCBs), which are segments of the sequence that are conserved among the isolates and free of internal rearrangements. Nucleotide sequence positions for each genome are indicated on a line above the LCBs. White boxes below the genomes for CnmeGV correspond to annotate ORFs, and red boxes correspond to homologous repeated regions (*hrs*). The height of the profile within each LCB corresponds to the average level of sequence conservation among the isolates in that region of the genome sequence. The nucleotide sequences of the *hr4*, *bro-A* and *bro-B* genes and their nearby regions were less conserved compared to other regions of the genome (The approximate positions of these three regions in the genome have been indicated).


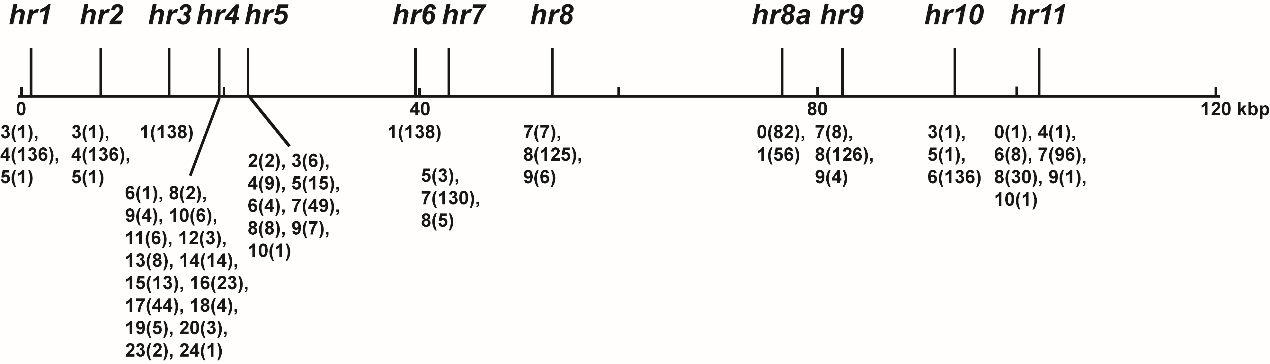


**Fig. S3:** The distribution and location of homologous repeated regions (*hrs*) in the CnmeGV genome. The *hrs* are indicated by vertical lines. On the left side of the brackets, the unit palindrome number of *hrs* is given. Inside the brackets, the number of isolates with this unit palindrome number is provided.


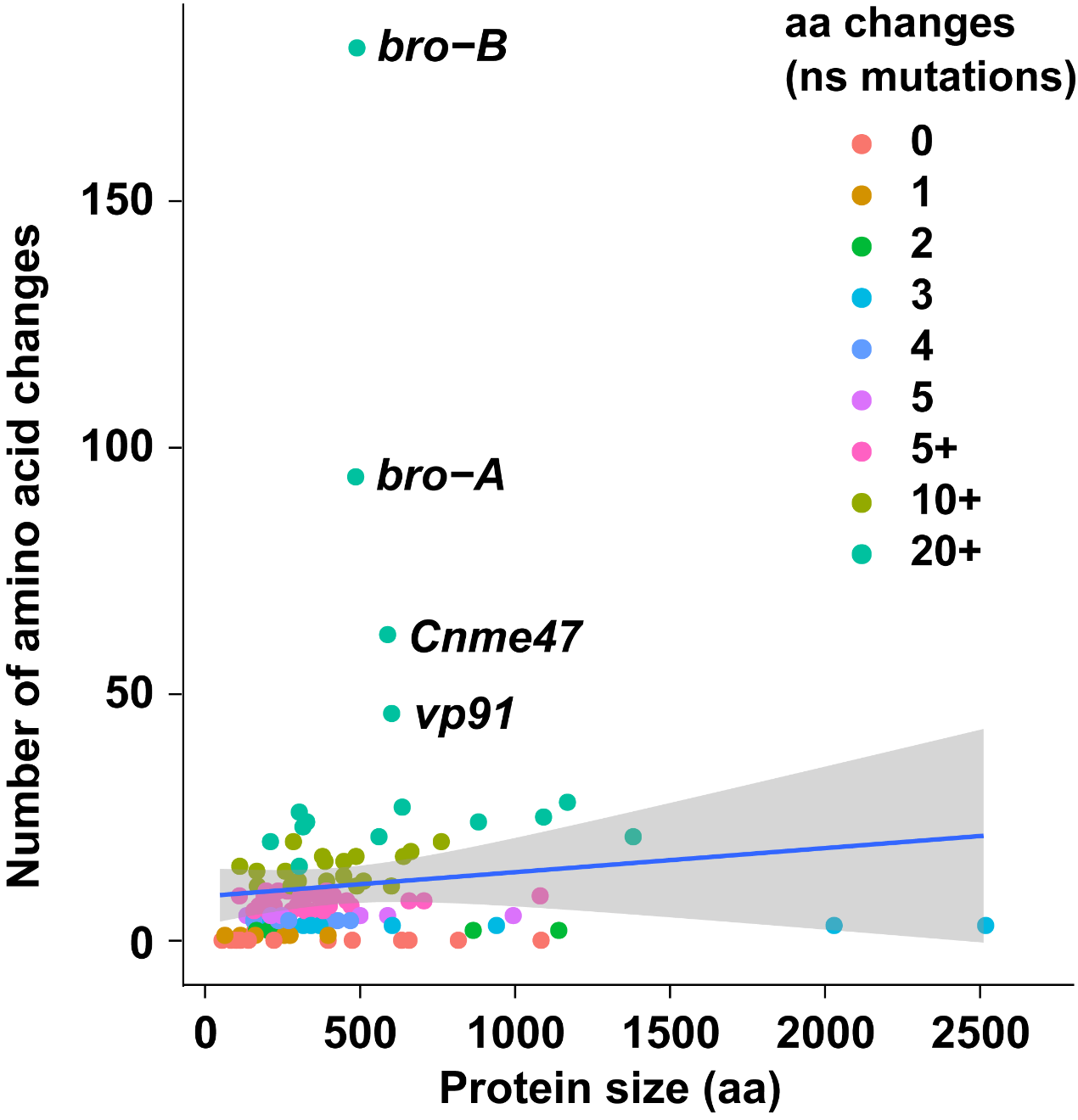


**Fig. S4:** The scatter plot showing the number of amino acid (aa) changes (nonsynonymous (ns) changes) as a function of protein size. A weak correlation (r = 0.08) was observed between the variables and a 95% confidence interval is displayed. The values that underlie the panels are provided in Section B of S2 Data.


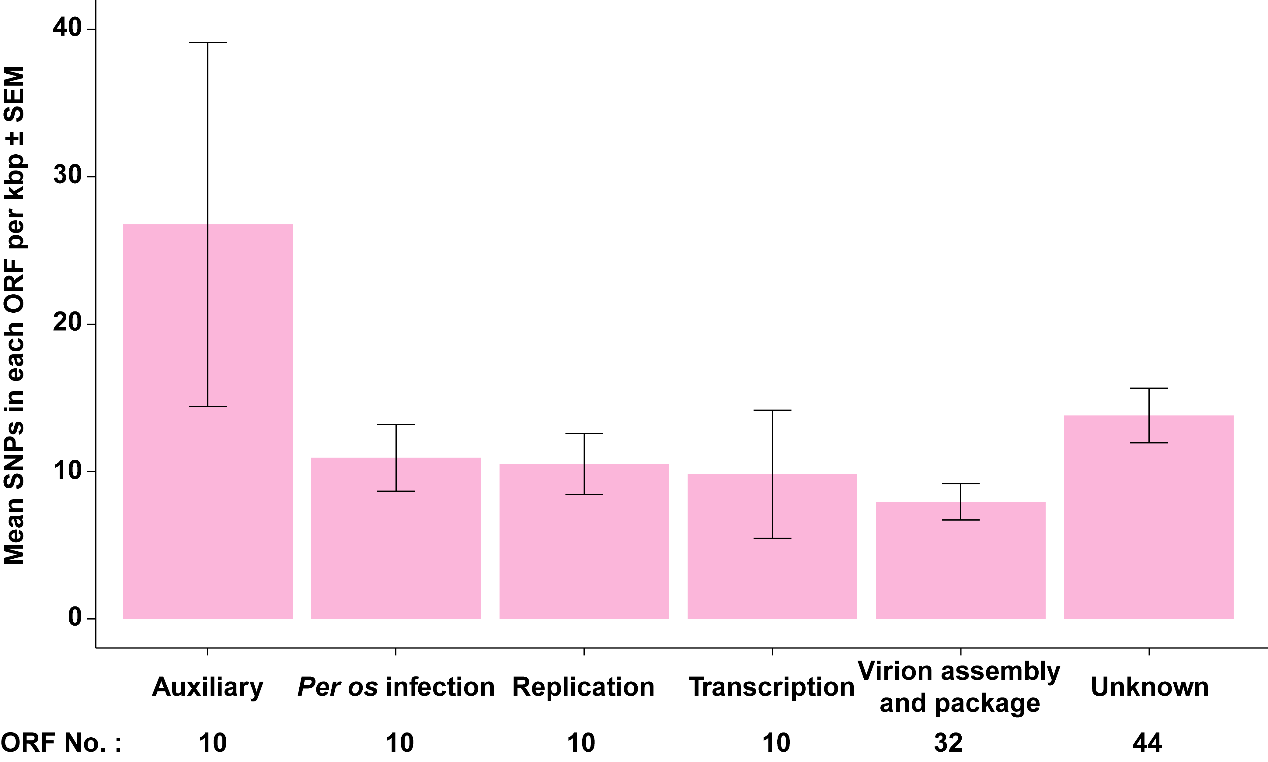


**Fig. S5:** The distribution of nonsynonymous changes in 116 shared Open reading frames (ORFs) that are grouped into six ORF functional categories. For each ORF, the number of nonsynonymous SNPs per kbp was determined. The vertical bar represents the average amount of SNPs in each ORF per kbp with the standard error (SEM). The values underlying the panels are provided in Section B of S2 Data.


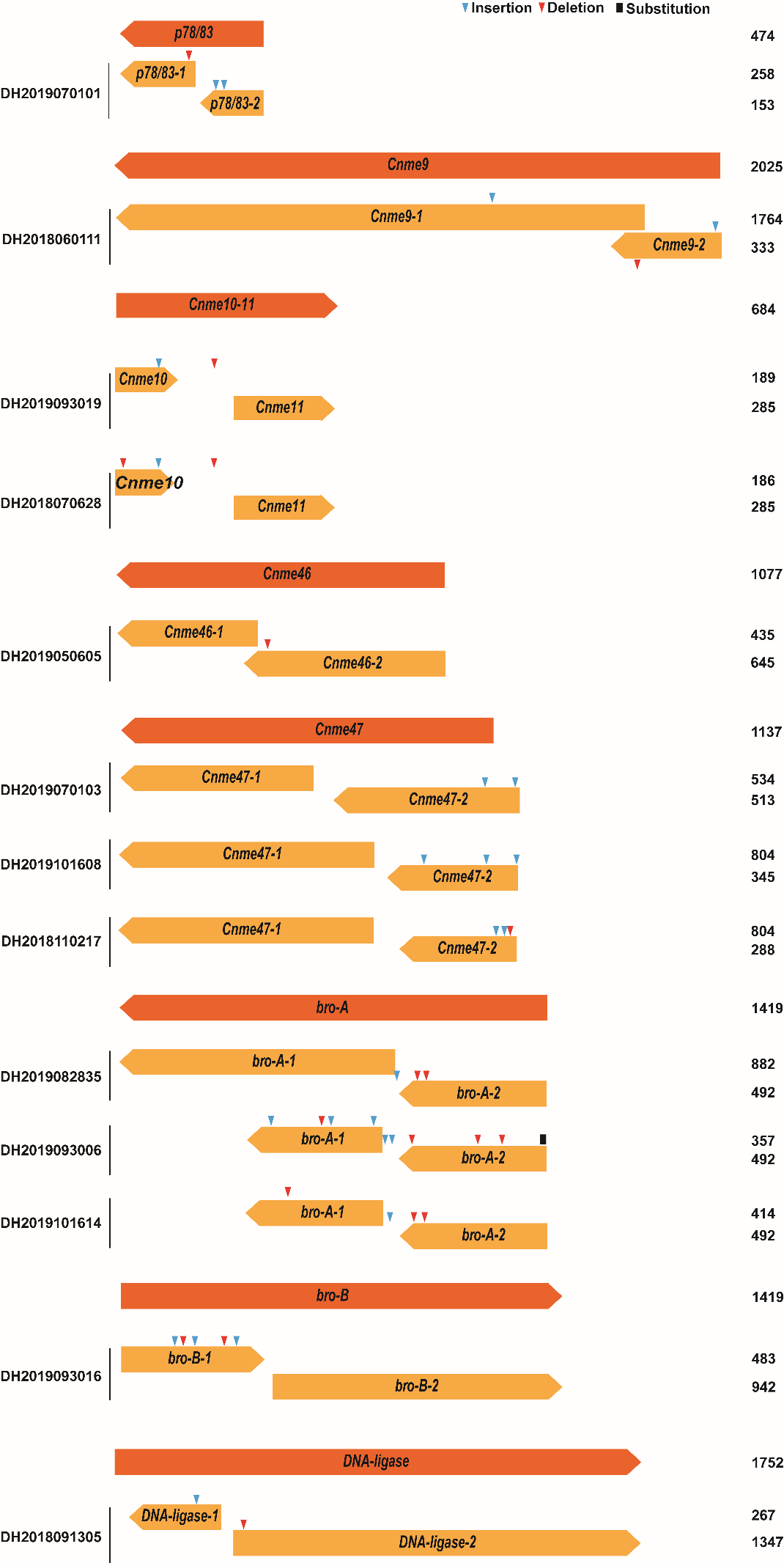


**Fig. S6:** Fission of genes in CnmeGV isolates. On the left side, the name of the isolate is displayed. On the right side, the nucleotide length of the Open reading frame (ORF) is shown. Orange arrows indicate ORFs that were previously annotated in the EPDH3 genome. Yellow arrows represent new ORFs that result from the fission event. Blue and red triangles respectively represent insertions and deletions. Black squares represent nucleotide substitutions. Detailed information is provided in Section D of S3 Data.

# C Calculating nucleotide diversity within individual isolates

As mentioned in the main text, to quantify the diversity of CnmeGV within individual isolates, average nucleotide diversity *π* is used. This is a standard population-genetic statistic employed to summarize diversity ([Kennedy and Dwyer, 2018](#_ENREF_27); [Nei and Li, 1979](#_ENREF_42)). Nucleotide diversity represents the probability that any two randomly selected alleles at a specific site in a population would be different if the population were in Hardy–Weinberg equilibrium. It is defined as:

$$\pi=1-\frac{1}{n}\sum_{j=1}^{n} \sum_{i=1}^{k_{j}} x_{ij}^{2}$$

Here, *π* represents the nucleotide diversity. In the equation, *k_j_* is the total number of allelic variants in the population at site *j*. *x_ij_* denotes the frequency of allele *i* at site *j*. And n is the total number of focal sites.

For this purpose, the reads were mapped to the reference genome EPDH3 by the MEM algorithm of BWA software ([Li and Durbin, 2009](#_ENREF_35)). The output “sam” files were converted to “bam” files using “samtools view” ([Li, 2011](#_ENREF_34); [Li et al., 2009](#_ENREF_36)). The “bam” files were sorted using “samtools sort”, and the sorted files were then converted to “mpileup” files using “samtools mpileup” with a minimum mapping quality of 20. Variant calling was carried out simultaneously using the function “mpileup2cns” in the program “VarScan” version 2.3.9 ([Koboldt et al., 2012](#_ENREF_28)). Variants were called at a minimum coverage of 100.

Since most sites are conserved both within and between individual isolates and provided little information on genetic variation, sites that were uniform within samples but segregated in more than 7 samples (approximately 5%) were identified as previously described ([Kennedy and Dwyer, 2018](#_ENREF_27)). By using·the "FindVariants/SNPs" program in Geneious software, 928 segregating sites at the between-isolate scale were identified from a comparison of the 138 CnmeGV consensus sequences.

We calculated the mean nucleotide diversity of the virus population within each isolate. The diversity of pathogens within the hosts presents three patterns (Patterns A-C) (Fig. 5A, 5B, and 5C in the main text). Nearly two-thirds (84, 60.9%) of the isolates likely consisted of only a single virus strain (Pattern A, Section E of S4 Data, Fig. S7A). More than one-third (54, 39.1%) of the isolates were a mixture of different virus strains (Patterns B and C, Sections F and G of S4 Data, Fig. S7B and S7C). In summary, the majority of natural isolates of CnmeGV from the same geographical origin are of a single genotype, whereas a small number are mixed genotypes. Genotype mixtures are frequently observed in baculovirus field isolates ([Larem et al., 2019](#_ENREF_31)). When viruses of different genotypes establish co-infection within the host, the virus may generate new genetic variations through recombination ([Gutiérrez et al., 2012](#_ENREF_15)).

We primarily conducted a relatively systematic sample collection of CnmeGV in Dahuai town in 2018 and 2019. During each rice-growing season, three waves of CnmeGV epidemics can occur, including the first wave (W1), the second wave (W2), and the third wave (W3). Except for the W1 of CnmeGV isolates from the late rice of 2018, which were not collected due to continuous heavy rains, samples of the three waves of CnmeGV epidemics were collected in each rice-growing season (Table S1). Among them, the W2 CnmeGV isolates from the early and late rice of 2018 were collected at two sampling time points (Table S1). We counted the median nucleotide diversity of CnmeGV isolates in each wave. Moreover, we deduced the median nucleotide diversity of the W1 CnmeGV isolates from the late rice in 2018 based on the average of the median nucleotide diversity of the W1 CnmeGV isolates from early rice of 2018 as well as early and late rice of 2019 (Table S1). We plotted the median nucleotide diversity of each wave of CnmeGV isolates using R (Fig. 5G in the main text). In the figure, based on the rice-growing conditions at that time, the occurrence time of the W1 isolates from the late rice in 2018 was estimated to be September. Additionally, the time of the W2 isolates from the late rice in 2018 which were collected on September 13^th^ and October 19^th^ was expressed as October. Table S1 provides the patterns of pathogens within the host in each epidemic wave in Dahuai town.

During a rice-growing season, the CnmeGV genomes exhibit higher heterozygosity levels in the initial epidemic wave compared to subsequent waves. This reflects that the CnmeGV epidemic experienced a transmission bottleneck and replication drift. New infections are typically initiated by small pathogen population sizes within hosts ([Gutiérrez et al., 2012](#_ENREF_15)). This leads to bottlenecks at the time of transmission, which may drive genetic drift. Pathogen population sizes within hosts can remain small for long periods after exposure ([Kennedy et al., 2014](#_ENREF_26)). In small populations, chance events like the timing of reproduction can have a significant impact on population growth ([Kot, 2001](#_ENREF_30)). This phenomenon is known as "demographic stochasticity" ([Kot, 2001](#_ENREF_30)). When the effects of demographic stochasticity are strong, chance plays a significant role. In such case, some virus strains may be able to replicate and survive by chance, whereas others go extinct. This provides a second source of genetic drift, which is referred to as "replicative drift".


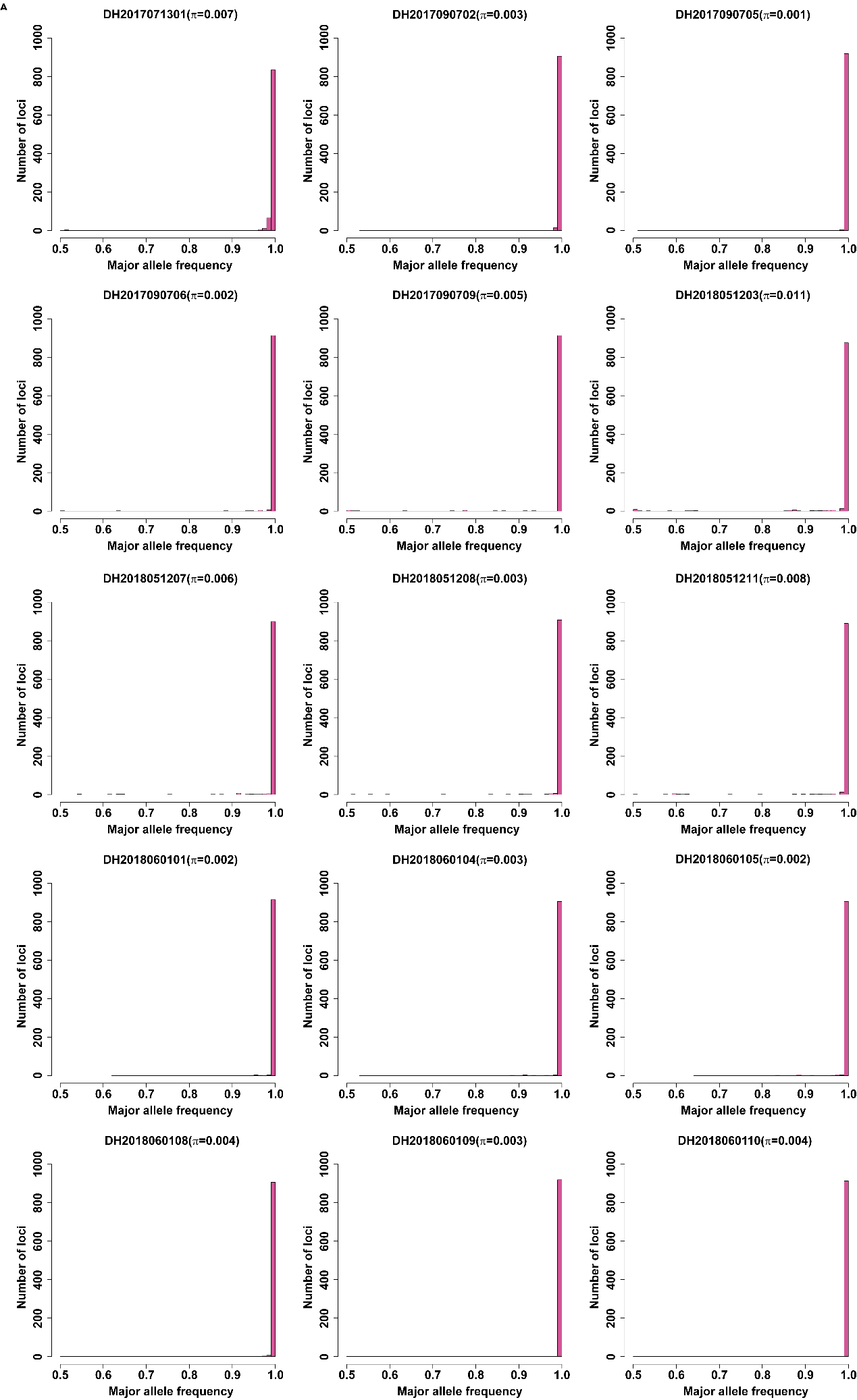


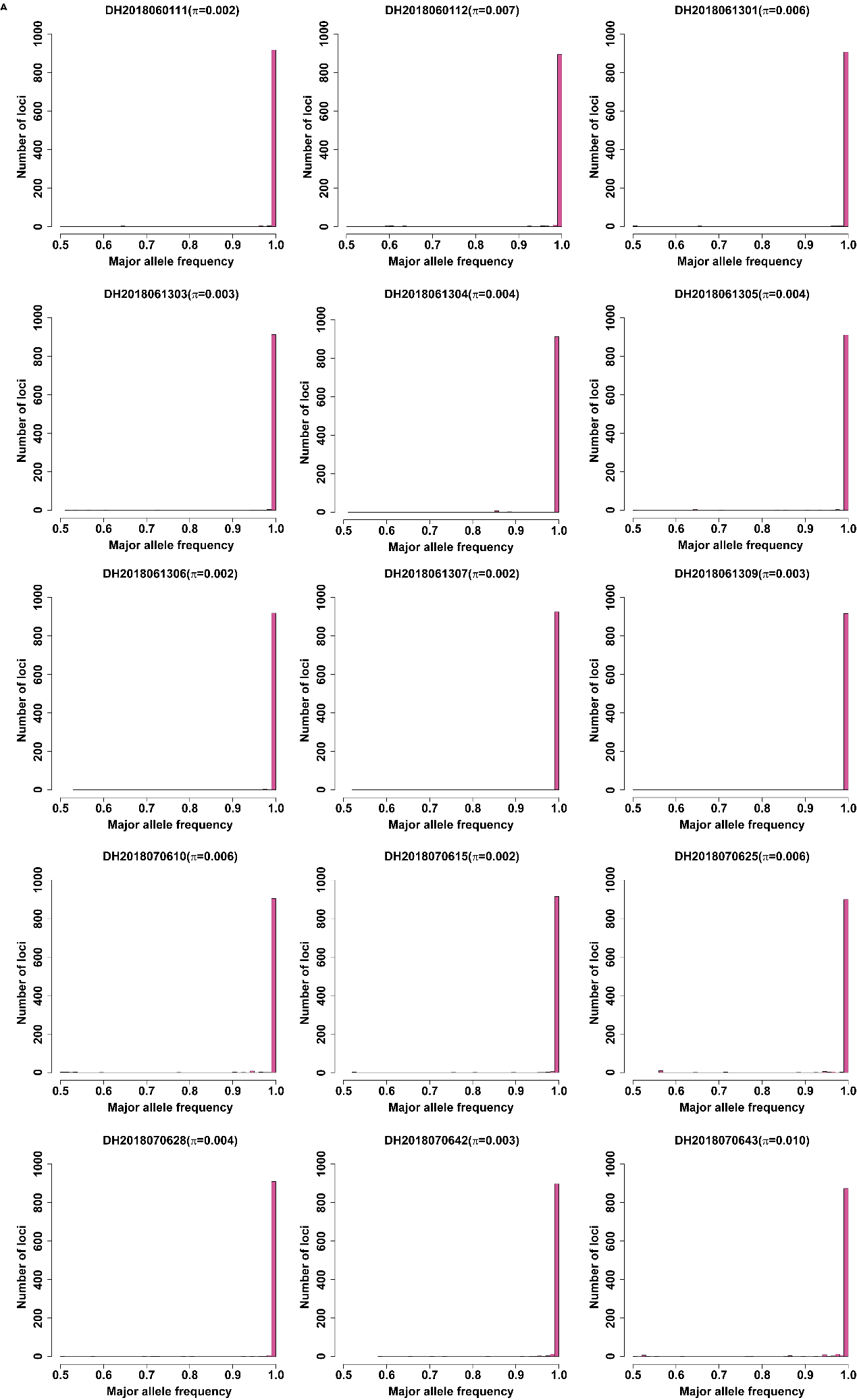


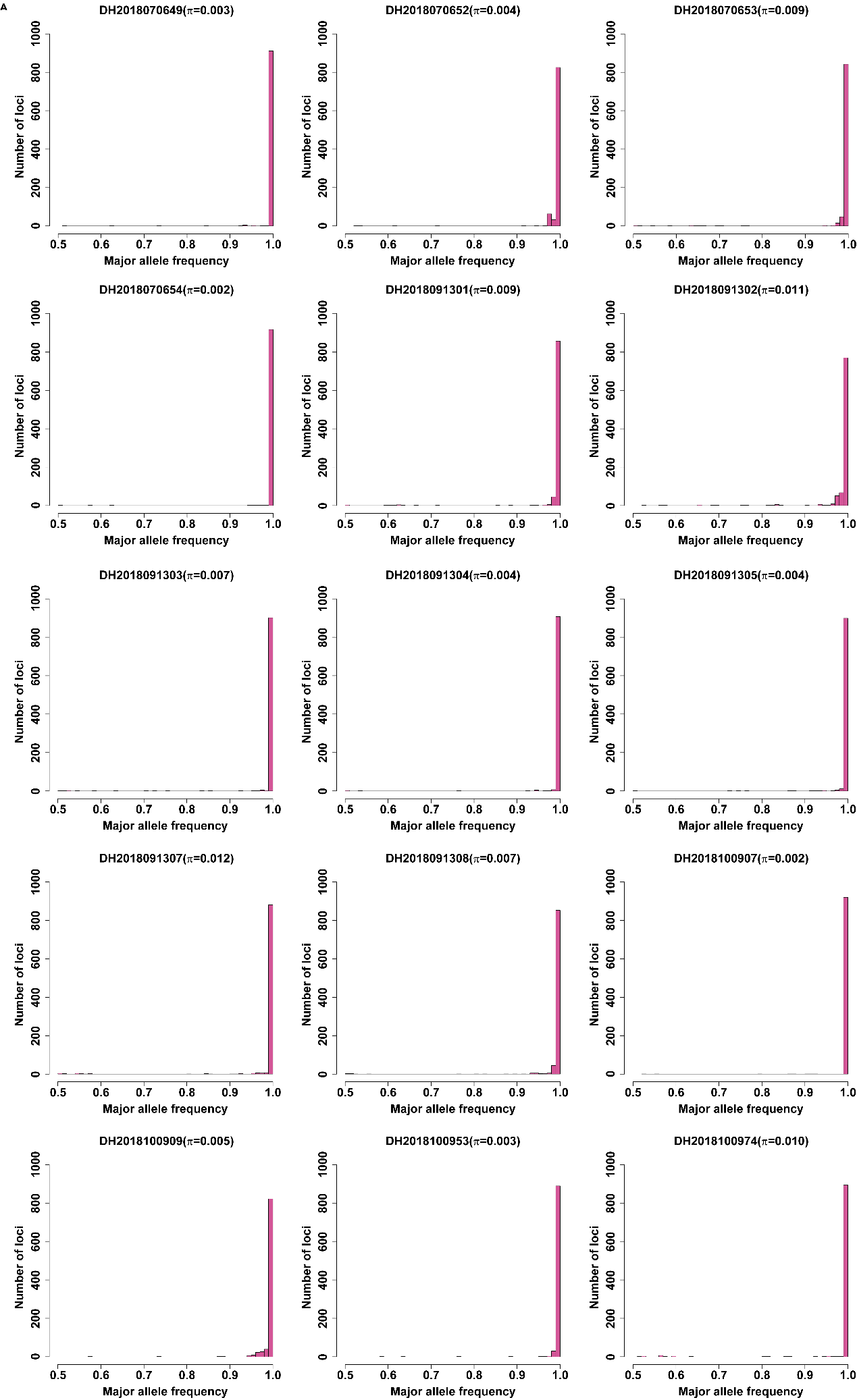


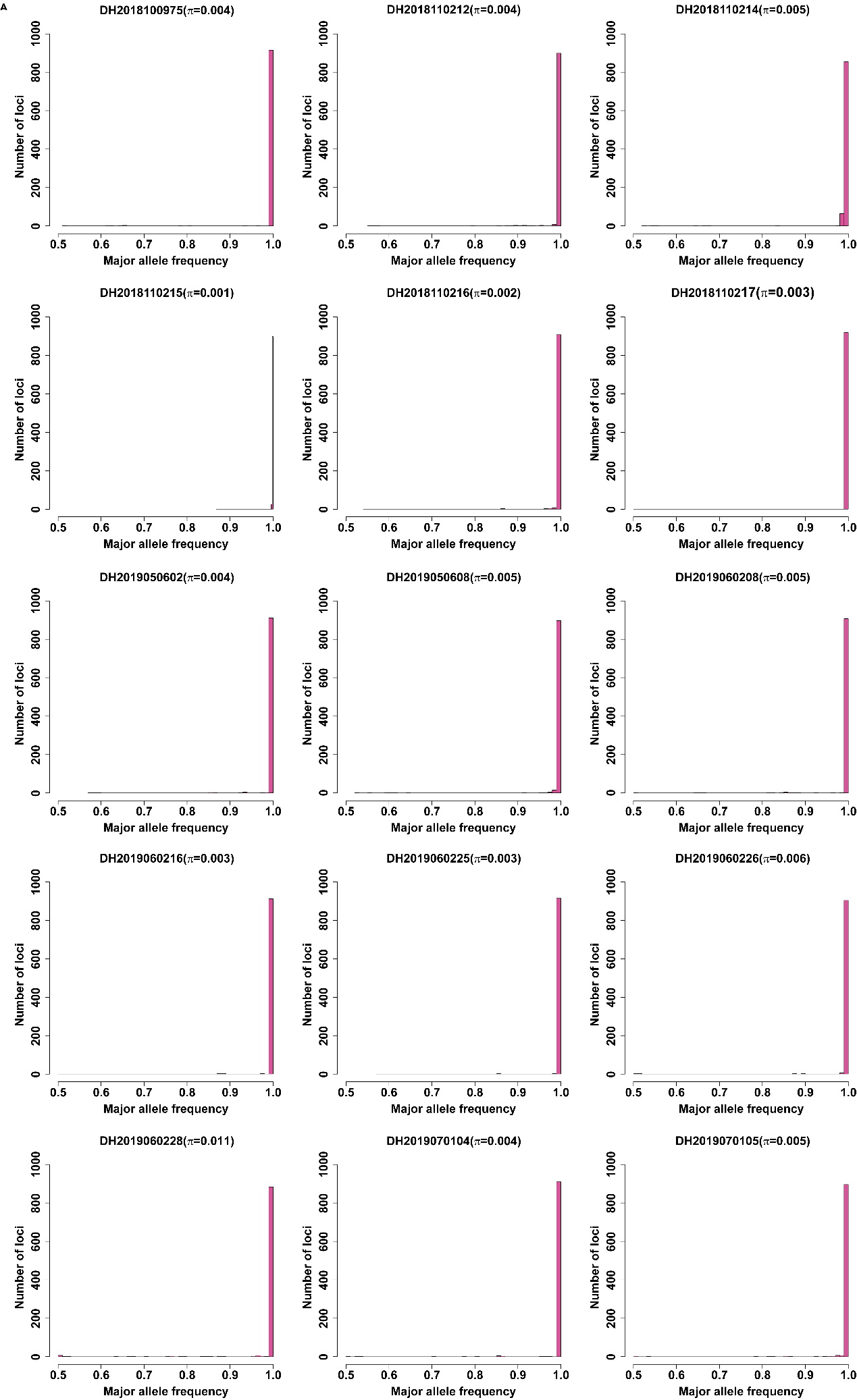


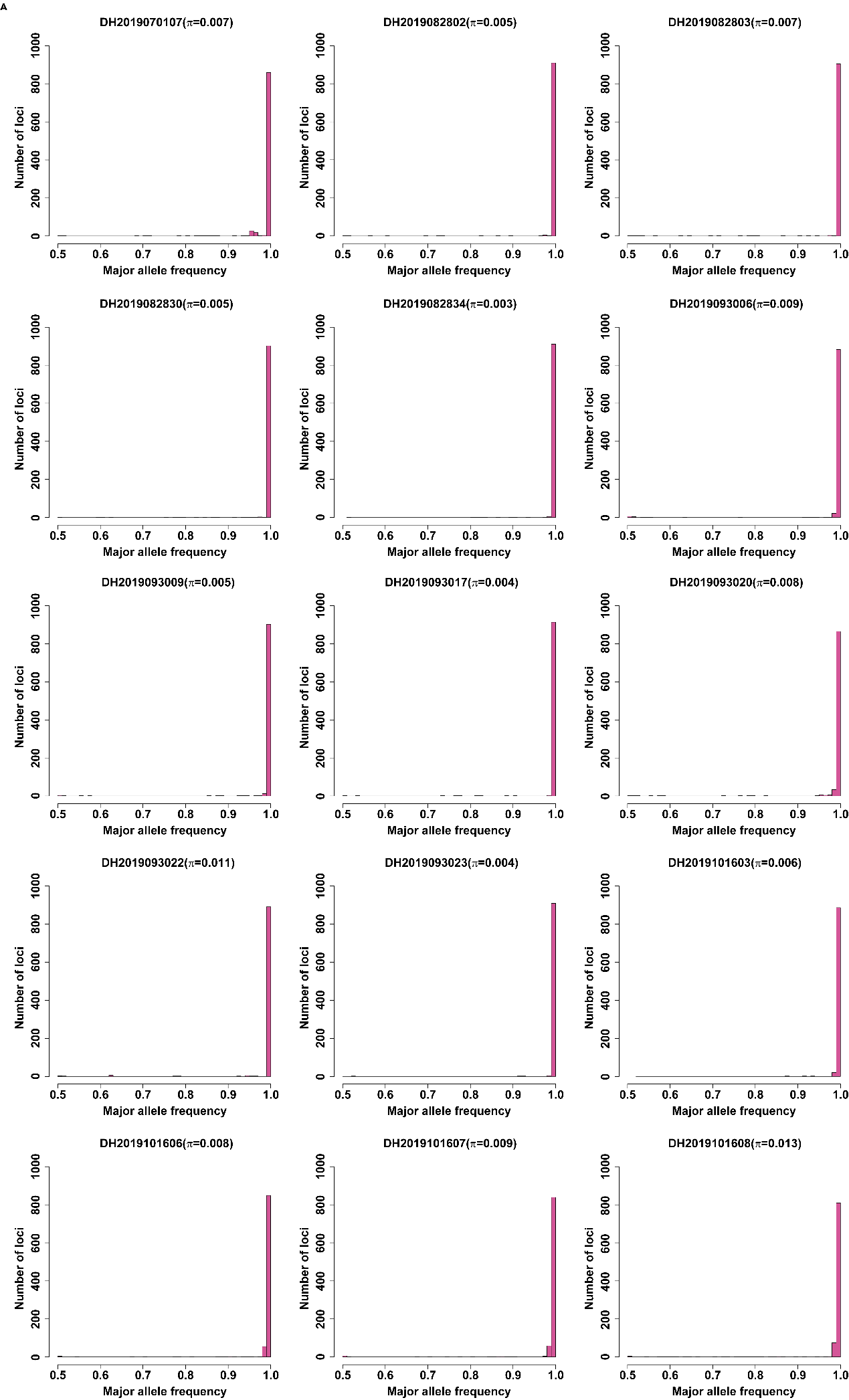


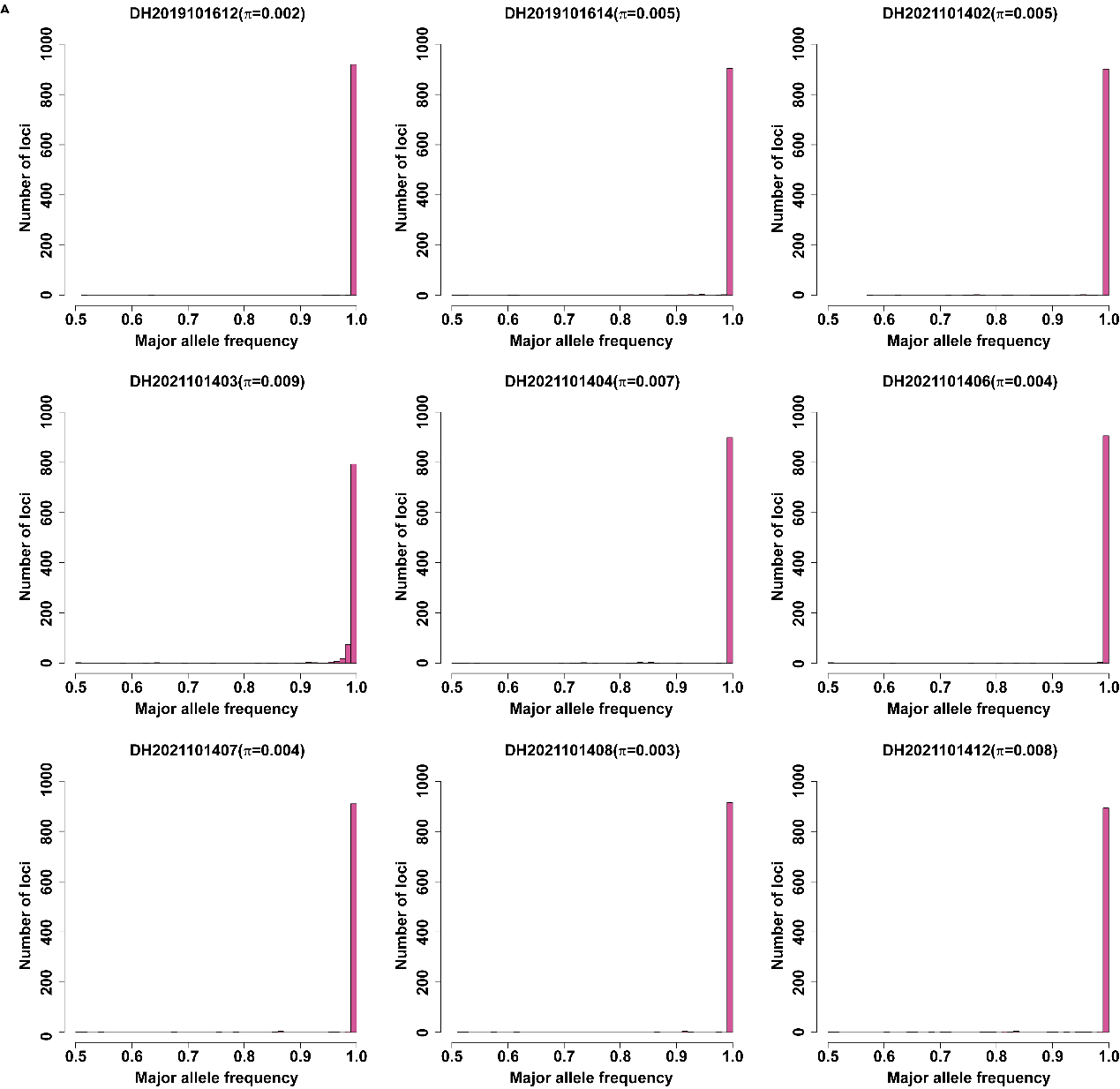


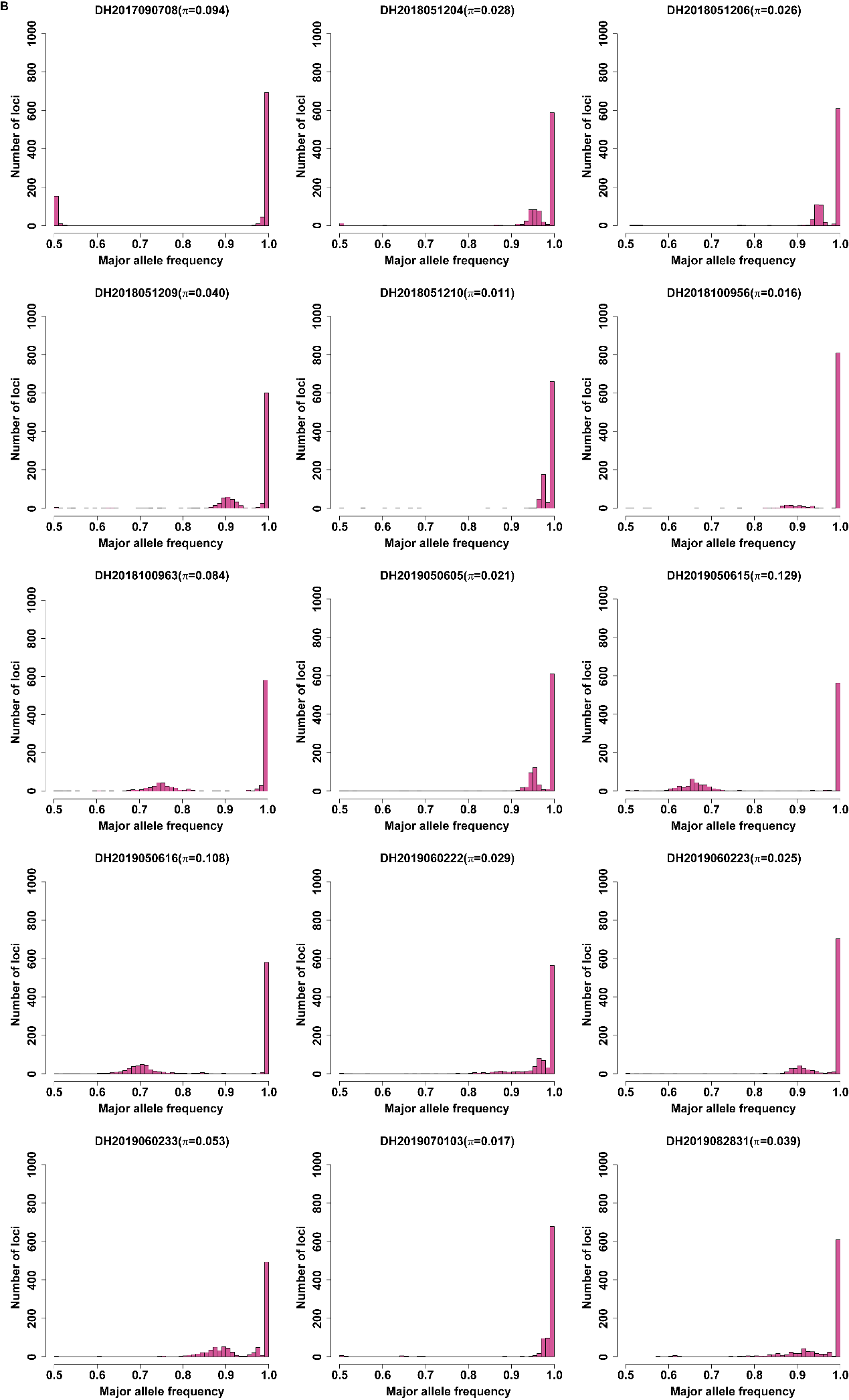


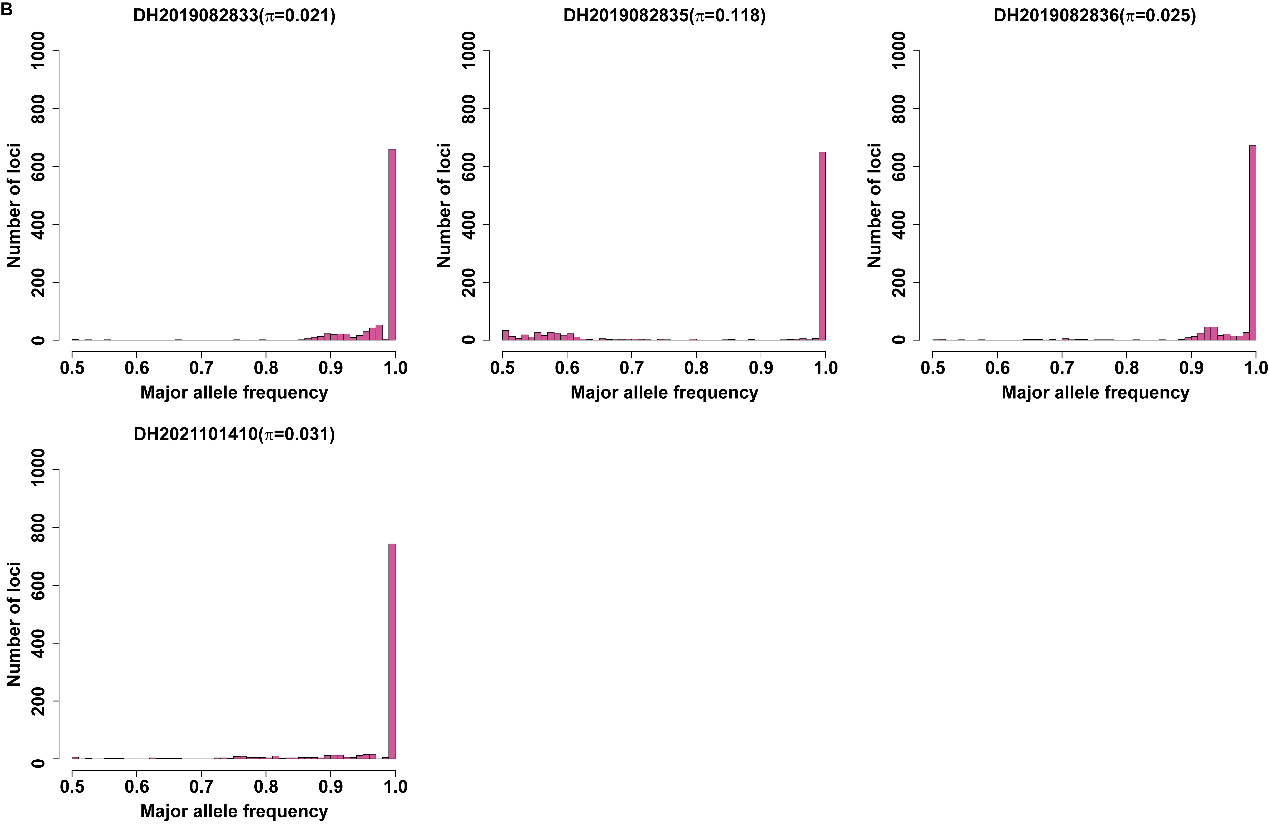


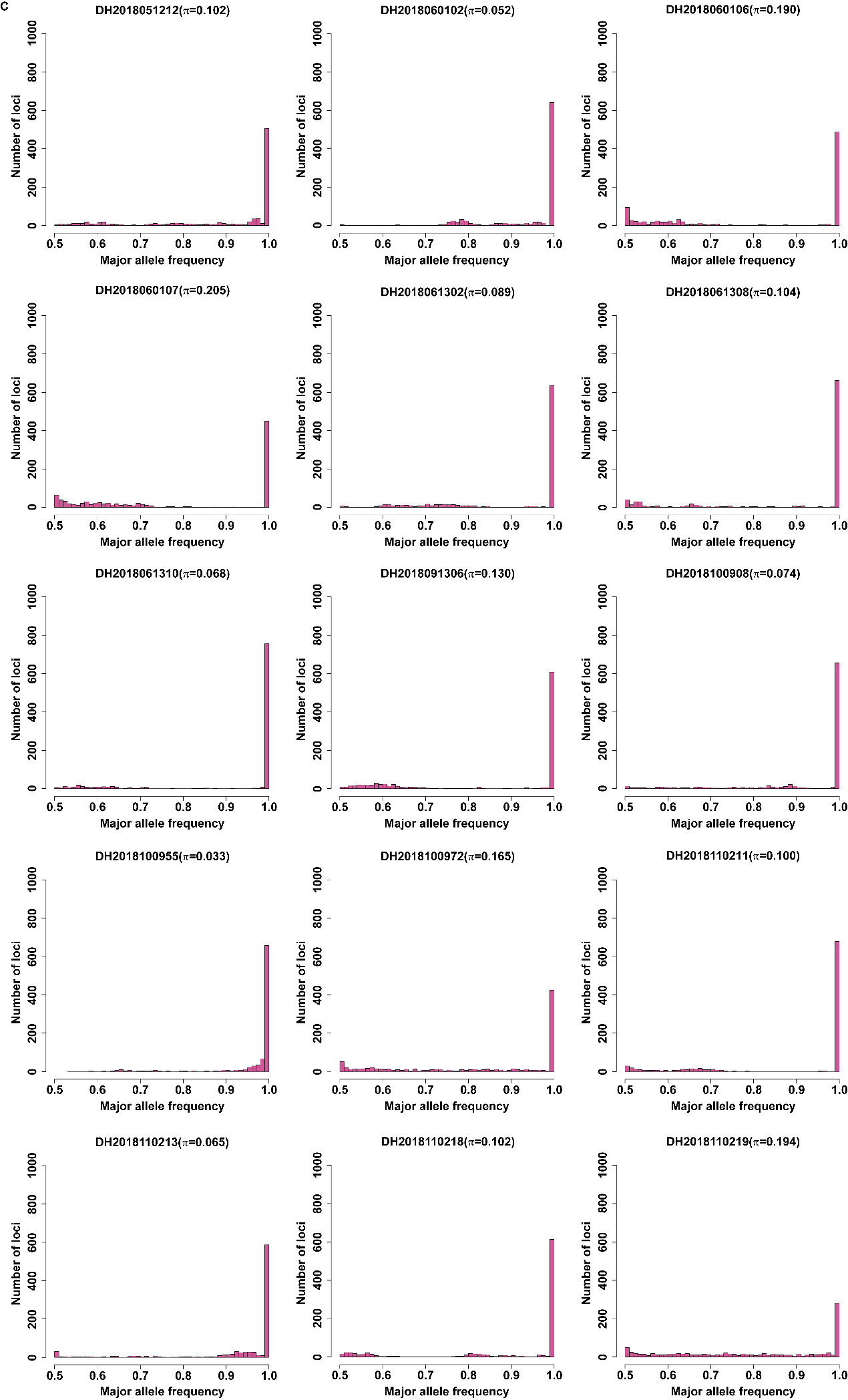


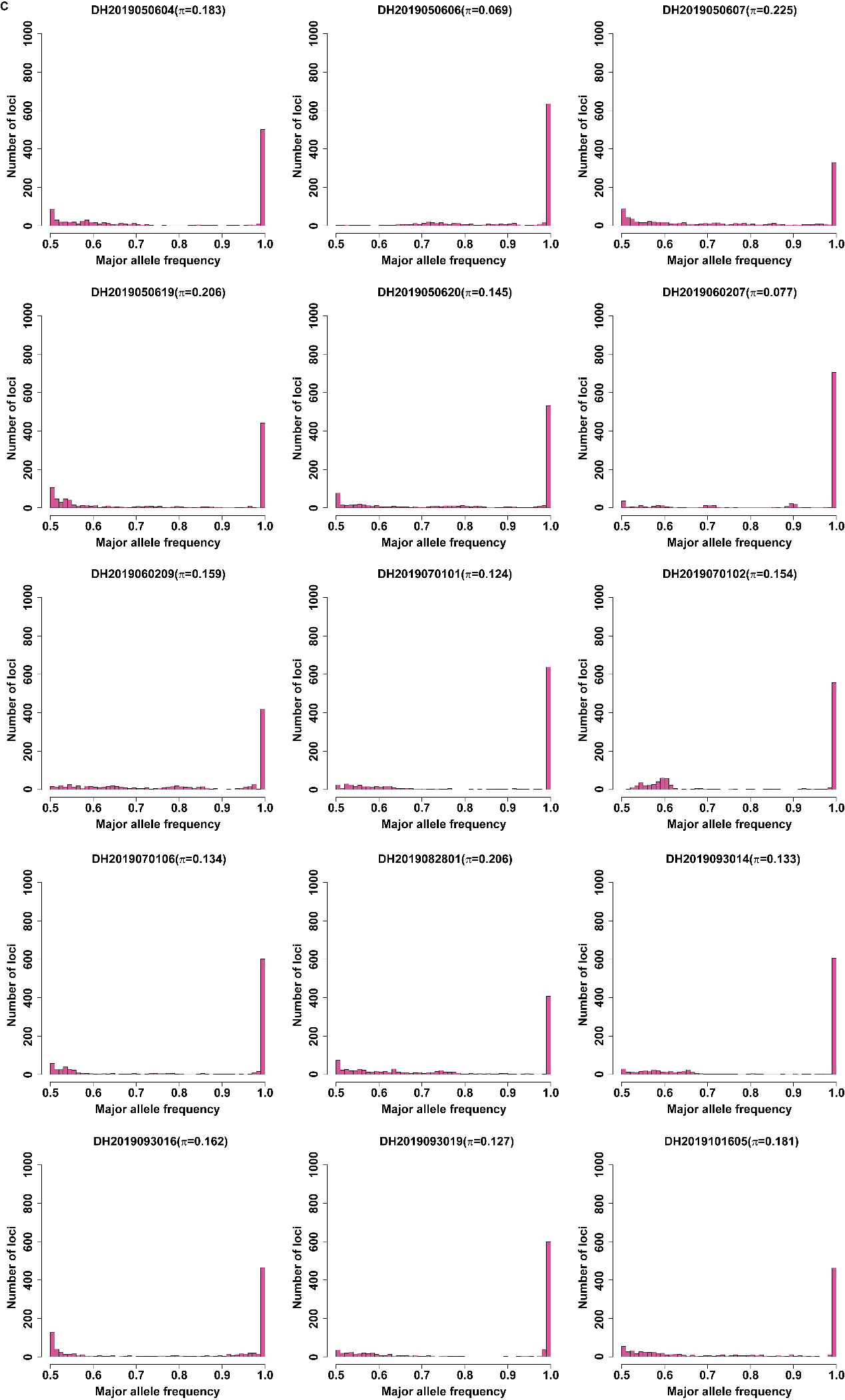


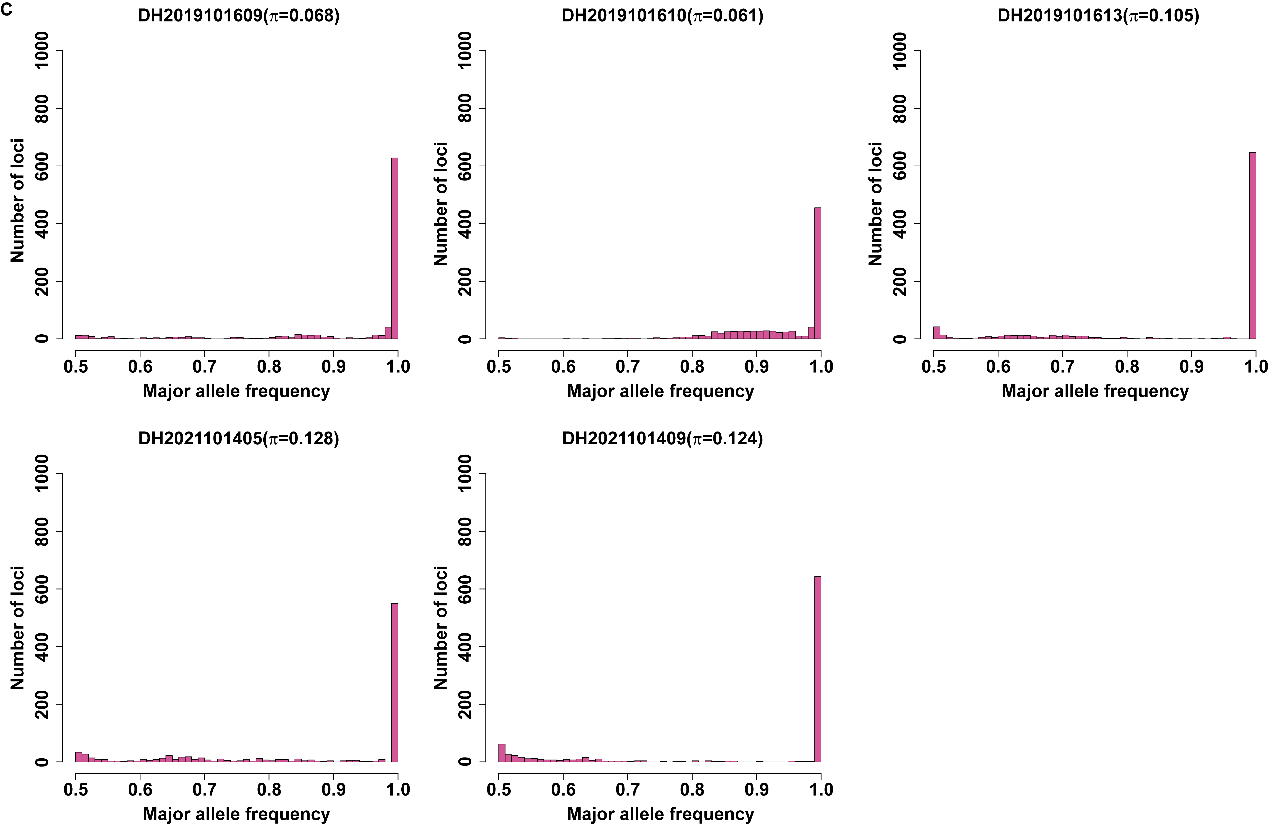


**Fig. S7:** Allele frequencies of pathogens within each host for 138 samples. Each plot represents the distribution of allele frequencies within a single individual at 928 segregating sites. Only the frequency of the most common allelic variant at each locus within that host. Each plot is marked with the sample ID. Additionally, the number in bracket on each plot represents the mean nucleotide diversity *π* within that specific host. There are three patterns (Patterns A-C) in the distribution of pathogen allele frequencies within each host. **A**, The absence of diversity in Pattern A implies that the virus population within these hosts consists of only a single virus strain. **B**, The presence of bimodal distributions in Pattern B indicates that these virus populations contain exactly 2 virus strains. **C**, The presence of high diversity yet lack of bimodality in Pattern C indicates that these virus populations likely consist of more than two virus strains. The plotted values are provided in Section A and E-G in S4 Data.

**Table S1:** Patterns of pathogens within the host in each epidemic wave in Dahuai town in 2018 and 2019.

| **Rice-growing season** | **Date** | **Epidemic waves** | **Median nucleotide diversity** | **Number of samples with A, B and C pattern (percentage)** | | |
| --- | --- | --- | --- | --- | --- | --- |
|  |  |  |  | **A** | **B** | **C** |
| 2018  early rice | May 12 | W1 | 0.011 | 4(44.4) | 4(44.4) | 1(11.2) |
|  | Jun. 1; 13 | W2 | 0.004 | 15(71.4) | 0(0.0) | 6(28.6) |
|  | Jul. 6 | W3 | 0.004 | 10(100.0) | 0(0.0) | 0(0.0) |
|  |  |  |  |  |  |  |
| 2018  late rice | Sep.^*^ | W1 | 0.026 | - | - | - |
|  | Sep. 13; Oct. 19 | W2 | 0.01 | 12(66.7) | 2(11.1) | 4(22.2) |
|  | Nov. 2 | W3 | 0.005 | 5(55.6) | 0(0.0) | 4(44.4) |
|  |  |  |  |  |  |  |
| 2019  early rice | May 6 | W1 | 0.119 | 2(20.0) | 3(30.0) | 5(50.0) |
|  | Jun. 2 | W2 | 0.018 | 5(50.0) | 3(30.0) | 2(20.0) |
|  | Jul. 1 | W3 | 0.017 | 3(42.9) | 1(14.3) | 3(42.8) |
|  |  |  |  |  |  |  |
| 2019  late rice | Aug. 28 | W1 | 0.021 | 4(44.4) | 4(44.4) | 1(22.2) |
|  | Sep. 30 | W2 | 0.009 | 6(66.7) | 0(0.0) | 3(33.3) |
|  | Oct. 16 | W3 | 0.011 | 6(60.0) | 0(0.0) | 4(40.0) |

Note: “-” indicates a missing value. “*” indicates an estimation. The occurrence time of the W1 isolates from the late rice in 2018 was estimated to be September. However, due to heavy rains at the beginning of tillering in 2018 and the lack of RLF, it was not possible to obtain the median nucleotide diversity of the W1 of CnmeGV isolates in this season. As a workaround, this value was taken as the average of that from the other three growing seasons. The W2 samples from both the early and late rice in 2018 were composed of isolates collected at two different sampling time points. “W1”, “W2”, and “W3” are used as designations for the first, second, and third wave of the CnmeGV epidemic respectively.

# D Calculating nucleotide diversity of CnmeGV in soil

Studies have shown that genetically diverse baculovirus OBs in soil can be transmitted to host larvae ([Williams et al., 2023](#_ENREF_50)). Soil samples (about 5 cm deep) were collected from paddy field in Dahuai town using the diagonal sampling method, in which samples were taken from three points along the diagonal transect of the field. The nucleotide diversity of CnmeGV in this soil was calculated. The samples were mixed evenly and a 10-g sample by wet weight was weighed. DNA was then extracted as described previously ([Moraes et al., 1999](#_ENREF_41)). Subsequently, the extracted DNA was purified using the LabServ Viral Total NA Kit. An Illumina sequencing library was constructed according to the instructions in the TruePrep® DNA Library Prep Kit V2 for Illumina (Vazyme).

The sequencing process generated 130 million 150-bp paired-end reads. However, the average sequencing coverage of CnmeGV was 62.2 ×. This could be attributed to the fact that the soil samples contained DNA from multiple species. The adapter sequence was trimmed using Cutadapt 1.15 ([Martin, 2011](#_ENREF_40)). After obtaining the bam file following the method in Section C, variants were searched in Geneious software. For calling variants, the following parameters were used: minimum coverage of 5, minimum variant frequency of 5%, and minimum quality of 30. A total of 898 SNPs were found (Section A of S5 Data). Out of these, 544 SNPs are in the 928 segregating sites described in Section D (Section B of S5 Data). Using these SNPs, the nucleotide diversity was calculated to be 0.384 according to the nucleotide diversity calculation formula in Section C. The nucleotide diversity of CnmeGV in the soil is higher than that of pathogens within each host and also higher than that between host.

# E Epidemiological data of CnmeGV

The incidence rate of RLF larvae (Section C of S5 Data) at each sampling stage was determined by calculating the ratio of the number of diseased larvae to the total number of larvae collected.

Covert infection of CnmeGV exists in RLF larvae. Covert infections are also known as inapparent, sublethal, silent or occult infections and are characterized by the absence of visible signs of disease ([Williams et al., 2017](#_ENREF_51)). To detect the presence of CnmeGV in the green transparent larvae, Nest-PCR was employed. Primers *gran*-F (outside) and *gran*-R (outside), as well as *gran*-F (inside) and *gran*-R (inside) (Table S2) were used to amplify the target *granulin* gene of CnmeGV. The homogenate of larvae that developed the disease after being infected with CnmeGV was used as the positive control, and ddH_2_O was used as the negative control.

Thirty green transparent larvae were randomly selected for Nest-PCR at each sampling stage. If there were less than thirty larvae available, all of them were detected. The positive rates of CnmeGV infection in RLF larvae at different stages in Dahuai town are provided in Section C of S5 Data.

Fig. S8 shows the fluctuations of the incidence of larvae and the CnmeGV positive rate of green-transparent larvae in the paddy field in Dahuai town at each time point in 2018 and 2019.

Soil is an important environmental reservoir of baculovirus OBs ([Hochberg, 1989](#_ENREF_21)). In the case of CnmeGV, since it does not contain a chitinase or a cathepsin gene ([Zhang et al., 2015](#_ENREF_54)), liquefaction or “melting” of the cadaver does not occur. As a results, OBs fall into the soil with the cadavers. Additionally, because OBs adhere strongly to soil particles, it is difficult to separate them by conventional techniques ([Evans et al., 1980](#_ENREF_11)). To detect the abundance of viral DNA in soil, 0.5 g of soil dried in an oven at 65 °C was weighed. DNA was extracted using a Mag-Bind Soil DNA Kit (OMEGA) according to the instructions. Then, real-time quantitative polymerase chain reaction (qPCR) was used to detect the abundance of viral DNA.

The average abundance of CnmeGV in the soil collected from the paddy field in Dahuai town using the plum blossom sampling method (i.e., collecting samples from the four corners and the center of the field) was determined to be 1.73 × 10^7^ copies/g soil. This is significantly higher than that the abundance in Shuikou town, Kaiping County, Guangdong Province, China, which was 3.78 × 10^3^ copies/g soil (Fig. S9). In Shuikou town, only covertly infected larvae with CnmeGV were found. The accumulation of CnmeGV in the Dahuai town paddy field was confirmed.


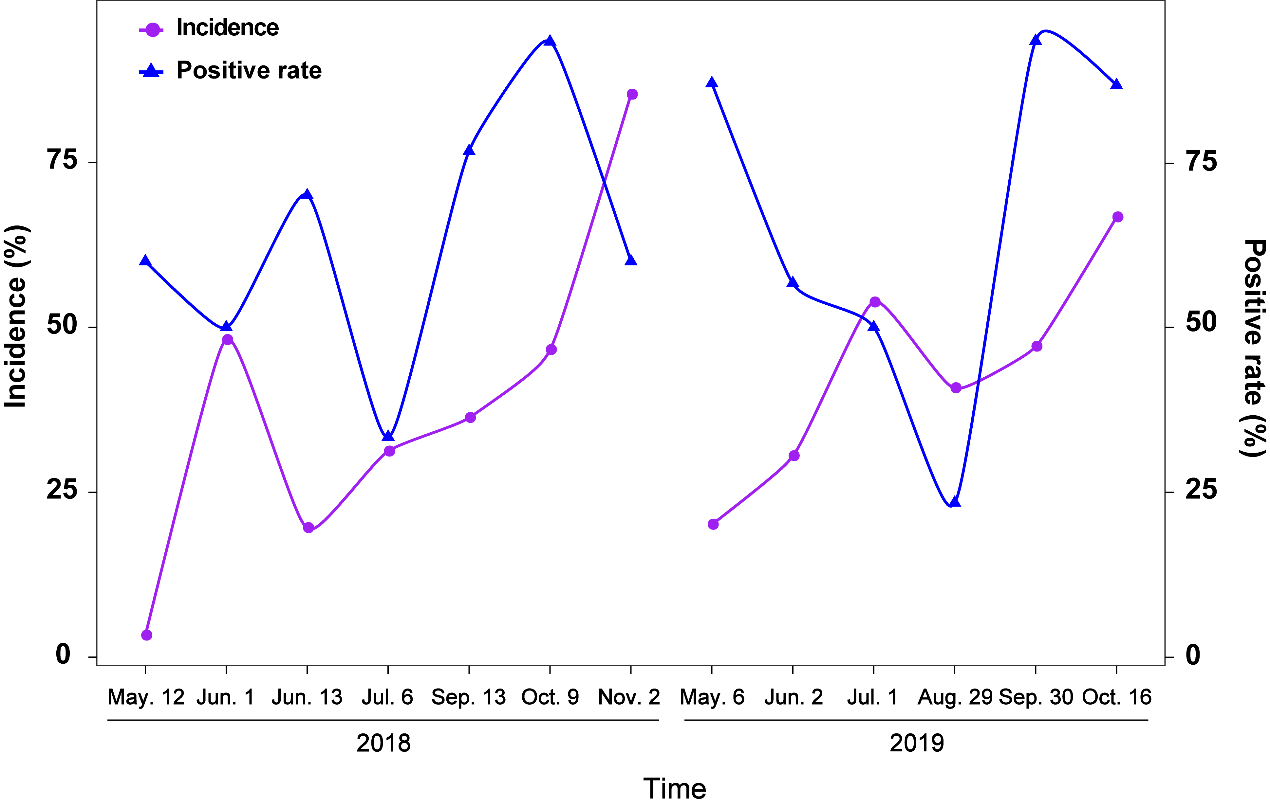


**Fig. S8:** The fluctuations in the incidence of larvae and the CnmeGV positive rate of green-transparent larvae in the paddy field of Dahuai town at each time point in 2018 and 2019. The purple curve shows the change in the incidence of larvae, and the blue curve represents the change in the CnmeGV positive rate of green-transparent larvae. The plotted values are provided in Section C of S5 Data.


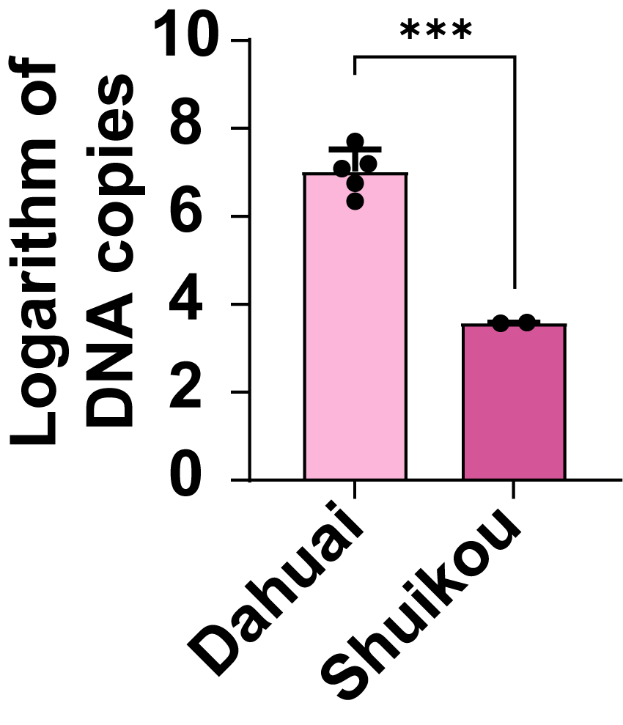


**Fig. S9:** Abundance of CnmeGV in paddy soils of Dahuai town and Shuikou town (Student’s t test, *** *p* < 0.001). Data are presented as mean values, and the error bars represent the standard deviation.

**Table S2: Primers for PCR**

| **Primer** | **Sequence (5**'**-3**'**)** | **Annealing temperature (°C)** | **Product size**  **(bp)** |
| --- | --- | --- | --- |
| *gran*-F | CATGATGCCGAGTATGAACCG | 60 | 163 |
| *gran*-R | CTTTTCCGCTCCAGTTGCACA |  |  |
| *gran*-F(outside) | ATCACTACGTCACAGCCGCCAC | 56 | 611 |
| *gran*-R(outside) | GACGGTAGAAGTAGGGCCAG |  |  |
| *gran*-F(inside) | TTGTGTAATCGACAACCAC | 56 | 467 |
| *gran*-R(inside) | TGTGTGCAATGTTGTCGGGTCC |  |  |

# References

Ayres, M.D., et al., (1994). ' The complete DNA sequence of Autographa californica nuclear polyhedrosis virus'. Virology 202, 586-605.

Benson, G., (1999). ' Tandem repeats finder: a program to analyze DNA sequences'. Nucleic Acids Res. 27, 573-580.

Bideshi, D.K., et al., (2003). ' Phylogenetic analysis and possible function of bro-like genes, a multigene family widespread among large double-stranded DNA viruses of invertebrates and bacteria'. J. Gen. Virol. 84, 2531-2544.

Boisvert, S., et al., (2010). ' Ray: simultaneous assembly of reads from a mix of high-throughput sequencing technologies'. J. Comput. Biol. 17, 1519-1533.

Bzymek, M., Lovett, S.T., (2001). ' Instability of repetitive DNA sequences: the role of replication in multiple mechanisms'. Proc. Natl. Acad. Sci. U. S. A. 98, 8319-8325.

Chen, Y.R., et al., (2013). ' The transcriptome of the baculovirus Autographa californica multiple nucleopolyhedrovirus in *Trichoplusia ni* cells'. J. Virol. 87, 6391-6405.

Cochran, M.A., Faulkner, P., (1983). ' Location of homologous DNA sequences interspersed at five regions in the baculovirus AcMNPV genome'. J. Virol. 45, 961-970.

Cui, Q., et al., (2013). ' Thymidylate kinase: an old topic brings new perspectives'. Curr. Med. Chem. 20, 1286-1305.

Darling, A.E., et al., (2010). ' progressiveMauve: multiple genome alignment with gene gain, loss and rearrangement'. PloS one 5, e11147.

De Jong, J.G., et al., (2005). ' Analysis of the Choristoneura fumiferana nucleopolyhedrovirus genome'. J. Gen. Virol. 86, 929-943.

Evans, H.F., et al., (1980). ' Methods for the quantitative assessment of nuclear-polyhedrosis virus in soil'. J. Invertebr. Pathol. 35, 1-8.

Fan, J., et al., (2020). ' Novel diversity and virulence patterns found in new isolates of Cydia pomonella granulovirus from China'. Appl. Environ. Microbiol. 86.

Gebhardt, M.M., et al., (2014). ' Baculovirus resistance in codling moth is virus isolate-dependent and the consequence of a mutation in viral gene *pe38*'. Proc. Natl. Acad. Sci. U. S. A. 111, 15711-15716.

Guarino, L.A., Summers, M.D., (1986). ' Functional mapping of a trans-activating gene required for expression of a baculovirus delayed-early gene'. J. Virol. 57, 563-571.

Gutiérrez, S., et al., (2012). ' Virus population bottlenecks during within-host progression and host-to-host transmission'. Curr. Opin. Virol. 2, 546-555.

Habib, S., Hasnain, S.E., (1997). ' A bifunctional baculovirus homologous region (*hr1*) sequence: enhancer and origin of replication functions reside within the same sequence element'. Curr. Sci., 658-666.

Hanada, K., et al., (2006). ' Radical amino acid change versus positive selection in the evolution of viral envelope proteins'. Gene 385, 83-88.

Harrison, R.L., et al., (2012). ' Genetic variation and virulence of Autographa californica multiple nucleopolyhedrovirus and Trichoplusia ni single nucleopolyhedrovirus isolates'. J. Invertebr. Pathol. 110, 33-47.

Harrison, R.L., et al., (2016). ' Geographic isolates of Lymantria dispar multiple nucleopolyhedrovirus: genome sequence analysis and pathogenicity against European and Asian gypsy moth strains'. J. Invertebr. Pathol. 137, 10-22.

Herniou, E.A., et al., (2003). ' The genome sequence and evolution of baculoviruses'. Annu. Rev. Entomol. 48, 211-234.

Hochberg, M.E., (1989). ' The potential role of pathogens in biological control'. Nature 337, 262-265.

Huang, Y.F., et al., (2019). ' Genomic sequencing of Troides aeacus nucleopolyhedrovirus (TraeNPV) from golden birdwing larvae (Troides aeacus formosanus) to reveal defective Autographa californica NPV genomic features'. BMC Genomics 20, 419.

Jacob, A., et al., (1973). ' Granulosis virus of the rice leaf roller, *Cnaphalocrocis medinalis* Güenée (Pyraustidae, Lepidoptera)'. Agric. Res. J. Kerala.

Kang, W., et al., (2006). ' The BRO proteins of Bombyx mori nucleopolyhedrovirus are nucleocytoplasmic shuttling proteins that utilize the CRM1-mediated nuclear export pathway'. Virology 350, 184-191.

Kearse, M., et al., (2012). ' Geneious Basic: an integrated and extendable desktop software platform for the organization and analysis of sequence data'. Bioinformatics (Oxford, England) 28, 1647-1649.

Kennedy, D.A., et al., (2014). ' Pathogen growth in insect hosts: inferring the importance of different mechanisms using stochastic models and response-time data'. Am. Nat. 184, 407-423.

Kennedy, D.A., Dwyer, G., (2018). ' Effects of multiple sources of genetic drift on pathogen variation within hosts'. PLoS. Biol. 16, e2004444.

Koboldt, D.C., et al., (2012). ' VarScan 2: somatic mutation and copy number alteration discovery in cancer by exome sequencing'. Genome Res. 22, 568-576.

Kool, M., et al., (1994). ' Identification of genes involved in DNA replication of the Autographa californica baculovirus'. Proc. Natl. Acad. Sci. U. S. A. 91, 11212-11216.

Kot, M., (2001). 'Elements of mathematical ecology'. Cambridge University Press.

Larem, A., et al., (2019). ' Elucidating the genetic diversity of Phthorimaea operculella granulovirus (PhopGV)'. J. Gen. Virol. 100, 679-690.

Leisy, D.J., et al., (1997). ' A mechanism for negative gene regulation in Autographa californica multinucleocapsid nuclear polyhedrosis virus'. J. Virol. 71, 5088-5094.

Levinson, G., Gutman, G.A., (1987). ' Slipped-strand mispairing: a major mechanism for DNA sequence evolution'. Mol. Biol. Evol. 4, 203-221.

Li, H., (2011). ' A statistical framework for SNP calling, mutation discovery, association mapping and population genetical parameter estimation from sequencing data'. Bioinformatics (Oxford, England) 27, 2987-2993.

Li, H., Durbin, R., (2009). ' Fast and accurate short read alignment with Burrows-Wheeler transform'. Bioinformatics (Oxford, England) 25, 1754-1760.

Li, H., et al., (2009). ' The sequence alignment/map format and SAMtools'. Bioinformatics (Oxford, England) 25, 2078-2079.

Li, L., et al., (2005). ' Complete comparative genomic analysis of two field isolates of Mamestra configurata nucleopolyhedrovirus-A'. J. Gen. Virol. 86, 91-105.

López-Ferber, M., et al., (2003). ' Defective or effective? Mutualistic interactions between virus genotypes'. Proc. Biol. Sci. 270, 2249-2255.

Lu, A., Miller, L.K., (1995). ' The roles of eighteen baculovirus late expression factor genes in transcription and DNA replication'. J. Virol. 69, 975-982.

Martin, M., (2011). ' Cutadapt removes adapter sequences from high-throughput sequencing reads'. EMBnet J. 17, 10-12.

Moraes, R.R., et al., (1999). ' Methods for detection of Anticarsia gemmatalis nucleopolyhedrovirus DNA in soil'. Appl. Environ. Microbiol. 65, 2307-2311.

Nei, M., Li, W.H., (1979). ' Mathematical model for studying genetic variation in terms of restriction endonucleases'. Proc. Natl. Acad. Sci. U. S. A. 76, 5269-5273.

O'Reilly, D.R., et al., (1992). 'Baculovirus expression vectors: a laboratory manual'. Oxford University Press.

Pang, Y., et al., (1981). ' A new granulovirus from naturally infected Asiatic rice leafroller, *Cnaphalocrocis medinalis* (Güenée)'. Microbiol. China, 103–104.

Pearson, M., et al., (1992). ' The Autographa californica baculovirus genome: evidence for multiple replication origins'. Science 257, 1382-1384.

Pearson, M.N., Rohrmann, G.F., (1995). ' Lymantria dispar nuclear polyhedrosis virus homologous regions: characterization of their ability to function as replication origins'. J. Virol. 69, 213-221.

Peng, K., et al., (2010). ' Baculovirus *per os* infectivity factors form a complex on the surface of occlusion-derived virus'. J. Virol. 84, 9497-9504.

Steinhaus, E.A., Marsh, G.A., (1962). ' Report of diagnoses of diseased insects 1951-1961'. Hilgardia 33(9), 349-490.

Wennmann, J.T., et al., (2017). ' Deciphering single nucleotide polymorphisms and evolutionary trends in isolates of the Cydia pomonella granulovirus'. Viruses 9.

Williams, T., et al., (2023). ' Presence of Spodoptera frugiperda multiple nucleopolyhedrovirus (SfMNPV) occlusion bodies in maize field soils of Mesoamerica'. Insects 14.

Williams, T., et al., (2017). ' Covert infection of insects by baculoviruses'. Frontiers in microbiology 8, 1337.

Zhang, H., et al., (2022). ' Genome analysis of Psilogramma increta granulovirus and its intrapopulation diversity'. Virus Res. 322, 198946.

Zhang, S., et al., (2014). ' Phylogenetic analysis and epidemiologic investigation of a Cnaphalocrocis medinalis granulovirus strain'. Environ. Entomol.

Zhang, S., et al., (2015). ' Genome sequencing and analysis of a granulovirus isolated from the Asiatic rice leafroller, *Cnaphalocrocis medinalis*'. Virol. Sin. 30, 417-424.

Zhou, J.-B., et al., (2012). ' Identification of a new Bombyx mori nucleopolyhedrovirus and analysis of its *bro* gene family'. Virus Genes. 44, 539-547.

Zuo, Y., et al., (2024). ' Genome comparison of long-circulating field CnmeGV isolates from the same region'. Virus Res. 345, 199390.
